# The impact of the *Deepwater Horizon* blowout on historic shipwreck-associated sediment microbiomes in the northern Gulf of Mexico

**DOI:** 10.1038/s41598-018-27350-z

**Published:** 2018-06-28

**Authors:** Leila J. Hamdan, Jennifer L. Salerno, Allen Reed, Samantha B. Joye, Melanie Damour

**Affiliations:** 1 University of Southern Mississippi, Ocean Springs, MS 39564 USA; 2 George Mason University, Manassas, VA 20110 USA; 3 U.S. Naval Research Laboratory, Stennis Space Center, Stennis, MS 39529 USA; 4 University of Georgia, Athens, GA 30602 USA; 5 Bureau of Ocean Energy Management, New Orleans, LA 70123 USA

## Abstract

More than 2,000 historic shipwrecks spanning 500 years of history, rest on the Gulf of Mexico seafloor. Shipwrecks serve as artificial reefs and hotspots of biodiversity by providing hard substrate, something rare in deep ocean regions. The *Deepwater Horizon* (DWH) spill discharged crude oil into the deep Gulf. Because of physical, biological, and chemical interactions, DWH oil was deposited on the seafloor, where historic shipwrecks are present. This study examined sediment microbiomes at seven historic shipwrecks. Steel-hulled, World War II-era shipwrecks and wooden-hulled, 19^th^ century shipwrecks within and outside of the surface oiled area and subsurface plume were examined. Analysis of 16S rRNA sequence libraries, sediment radiocarbon age data, sedimentation rates, and hydrocarbons revealed that the German U-boat *U*-166 and the wooden-hulled sailing vessel known as the Mardi Gras Wreck, both in the Mississippi Canyon leasing area, were exposed to deposited oil during a rapid sedimentation event. Impacts to shipwreck microbiomes included a significant increase in Piscirickettsiaceae-related sequences in surface sediments, and reduced biodiversity relative to unimpacted sites. This study is the first to address the impact of the spill on shipwreck-associated microbiomes, and to explore how shipwrecks themselves influence microbiome diversity in the deep sea.

## Introduction

The *Deepwater Horizon* (DWH) spill of 2010 resulted in an uncontrolled release of 5 million barrels of oil^1^. Approximately 47 thousand barrels of the dispersants Corexit 9500 and 9527 were applied at the surface and the wellhead to break up slicks and retain oil below the surface in dispersed plumes^2^. The spill introduced oil and chemical dispersants into marine environments. Together, this caused a deep-water infusion of pollutants^3^ that persisted for months. Discharged pollutants from the spill were deposited to the seabed, in association with residual dispersant and pelagic biomass, via marine oil snow across 8,400 km^2^ ^1,3–6^.

An estimated 11–30% of oil from the DWH spill remains unaccounted for^4,7^. It is possible that the ‘missing oil’ was deposited on the seafloor or to coastal beaches and marshes where it remains difficult to track. The mechanisms for transfer of discharged oil to the seafloor was through interaction with suspended particulate organic matter and inorganic minerals in the water column^4,5^, and through bacterio- and phytoplankton production of exopolymeric substances during oil degradation^3,5^. Both processes reflect the “marine oil snow sedimentation and flocculent accumulation” (MOSSFA) which efficiently transfers oil from the water column to the seabed. Oil transported to the seafloor via these mechanisms occurred during massive sedimentation events. Understanding the spatial deposition and longevity of oil on the seafloor remains an important challenge in understanding the long-term consequences of the spill.

Using data from the Natural Resource Damage Assessment (NRDA), Stout *et al*.^6^ described a footprint of DWH spill-derived hydrocarbons across a 2280 km^2^ area of seafloor in the Mississippi Canyon leasing area, a subset of the area noted above. The footprint was still evident in 2014. DWH-derived pollutants impacted fauna, including cold water corals, in deep-sea benthic habitats^8,9^. Given the magnitude of the incident, and rapid delivery of hydrocarbons to benthic environments, it is likely that deep-water habitats remain impacted.

**Figure 1:**
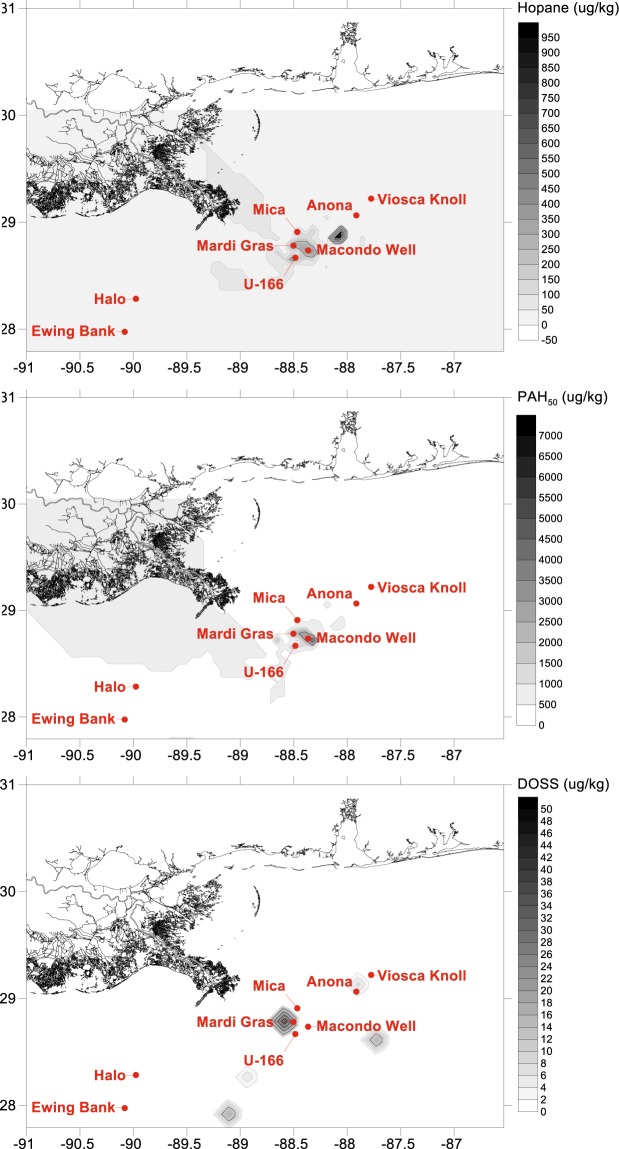
Study sites, and concentrations of seafloor hopane, PAH_50_, and dioctyl sodium sulfosuccinate (DOSS), a marker compound for Corexit 9500 residue. Grid data from NRDA sediment (top 10 cmbsf) collection efforts between 2010 and 2014. Data downloaded from the NOAA DIVER explorer (https://www.diver.orr.noaa.gov). Grids and maps were created using NRDA data and BOEM site coordinates in Surfer v.14 (Golden Software).

Within the footprint^6^ is an area extending ~16 km southwest of the Macondo well described as having an “acute footprint” from the spill. In this area, where sediment hopane concentrations indicative of DWH oil deposits exceed 800 ng g^−1^ ^1,6^, are three historic shipwrecks (Fig. 1)^10^. The shipwrecks are the steel-hulled, German U-boat *U*-166 and two wooden-hulled, 19^th^ century shipwrecks known as the Mica Wreck and the Mardi Gras Wreck. The proximity of these shipwrecks to the Macondo well, and location within the DWH spill footprint, raises questions about the spill’s impact on biota on and surrounding these artificial reefs, and the physical integrity of the shipwrecks.

The impacts of the spill on historic shipwrecks and the surrounding seafloor, are largely unknown. Microbial communities on shipwrecks (biofilms) and in the surrounding seafloor play important roles in shipwreck preservation and degradation, and in recruitment of macro-organisms to artificial reefs^11,12^. Accordingly, the purpose of this study was to document the lasting effects of the DWH spill on shipwrecks and the surrounding environment using an integrated microbiological, geochemical, and archaeological approach.

## Results

### Study sites

**Figure 2:**
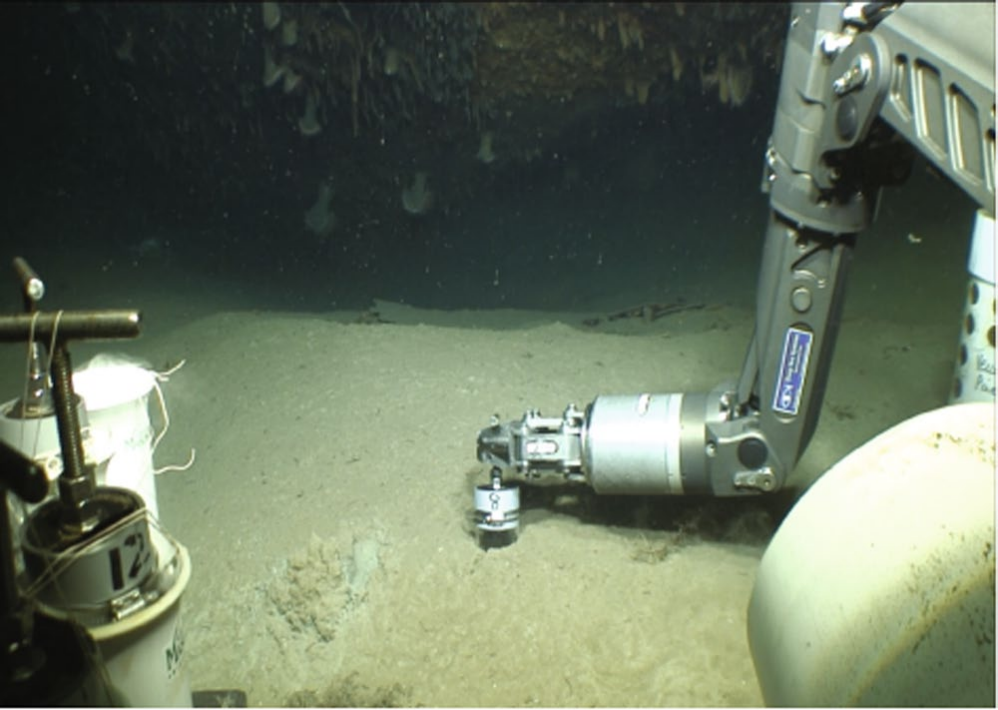
Sediment push core samples collected with the *Global Explorer* remotely operated vehicle (GEX-ROV). The GEX-ROV manipulator arm and core tube are in foreground. The *Anona* shipwreck’s stern is in the background. Cores were collected within 2 m of each shipwreck and at distances of 75–200 m away, depending on the site. Photograph taken by the GEX-ROV, operated by Deep Sea Systems International during expedition PE14–15, and provided by the Bureau of Ocean Energy Management.

Seven historic shipwrecks were selected based on depth and location relative to the Macondo Wellhead (Table 1 and Fig. 1), and the existence of pre-Macondo archaeological investigations to provide a baseline. Sites were selected from the Bureau of Ocean Energy Management (BOEM) and Bureau of Safety and Environmental Enforcement (BSEE) shared shipwreck database^13,14^. In three areas located within 20 km of the wellhead, 50–100 km northeast, and 100–150 km southwest, one steel-hulled, World War II-era (1942–1944) shipwreck and one or two 19^th^ century wooden-hulled, copper-sheathed shipwrecks were selected. These areas were selected based on distance and the surface oiling extent during the 87-day discharge (https://erma.noaa.gov/gulfofmexico/erma.htm). This approach distinguished three areas: heavily impacted (*U*-166, Mica and Mardi Gras Wrecks), moderately impacted (*Anona* and Viosca Knoll Wreck), and reference – outside of the surface oiled area and subsurface plume (*Halo* and Ewing Bank Wreck)^13,14^. Sediment samples from these sites were obtained within 2 m of each shipwreck, and 100–200 m away from each shipwreck (Fig. 2). Seafloor contamination of moderately and heavily impacted sites, and a lack of Macondo influence at reference sites, is supported by NRDA data in the NOAA Data DIVER repository (https://www.diver.orr.noaa.gov). Data for hopanes, the 50 most abundant polycyclic aromatic hydrocarbons (PAHs), and dioctyl sodium sulfosuccinate (DOSS) from the NRDA dataset from the study areas underscores the differences between sites (Fig. 1).

**Table 1:** Sampling locations and shipwreck descriptions.

Site Name	Vessel Type	Depth (m)	Site Type	Lease Area	Hull Material	L × W (ft)	Near site collection	Away from site collection	Date Lost	Prior Survey Years
*U*-166	U-Boat	1450	Heavily Impacted	Miss. Canyon	Steel	bow 65 × 22; stern 180 × 22*	starboard bow	200 m W of bow	Jul-42	2001 2003 2004 2009 2010
*Halo*	Tanker	140	Reference	Grand Isle	Steel	436 × 66	port aft	200 m E	May-42	2004
*Anona*	Steam Yacht	1250	Moderately Impacted	Viosca Knoll	Steel	136 × 17	stern	none	Jun-44	1995 2002 2006
Mardi Gras	Sailing	1220	Heavily Impacted	Miss. Canyon	Wood - Copper Sheathed	48 × 14	forward of weapons chest	100 m E	19th century	2004 2005 2009
Mica	Sailing	800	Heavily Impacted	Miss. Canyon	Wood - Copper Sheathed	65 × 20	port stern	100 m S	19th century	2001 2003
Viosca Knoll	Sailing	600	Moderately Impacted	Viosca Knoll	Wood - Copper Sheathed	140 × 52	port aft	200 m SW	19th century	2004 2006 2009
Ewing Bank	Sailing	600	Reference	Ewing Bank	Wood - Copper Sheathed	148 × 39	bow	100 m E	19th century	2006 2007 2008 2009

*Sampling occurred at bow section of *U*-166 shipwreck.

### Microbiome composition

On average, 7,594 and 34,429 sequences with median length of 442 base pairs were obtained per sample for bacteria and archaea respectively (Table 2). Despite a lower sequence count, operational taxonomic units (OTU) count and Shannon diversity was greater for bacteria than archaea. The Chao 1 reveals that bacteria and archaea were under-sequenced by 30% and 21% respectively (Supplementary Table S1). The Good’s coverage index was calculated to evaluate the impact of sample size on results. The index averaged 0.81 and 0.93 for bacteria and archaea, respectively, indicating excellent coverage of archaeal communities, but under-sequencing of bacterial singletons. Bacterial Shannon diversity did not differ in surface and bottom sediments collected within 2 m of shipwrecks. However, in samples away from shipwrecks, diversity was generally lower near the surface of cores (Table 2). Archaeal diversity was lower in surface vs. deep sediments both near and away from shipwrecks. Shannon diversity was higher in samples collected at the surface, near shipwrecks, compared to away from shipwrecks, except at the Mardi Gras Wreck. Analysis of similarity (ANOSIM) revealed that microbiomes near and away from shipwrecks differed significantly (p ≤ 0.05) at moderately impacted and reference sites, but not at heavily impacted sites (Table 2).

**Table 2:** Descriptive statistics for surface (0–10 cm below seafloor, cmbsf) and deep (11–22 cmbsf) sediment samples collected proximate to and away from shipwrecks.

Site	Depth (cmbsf)	Near Shipwreck (1–2 m)				Away from Shipwreck (100–200 m)				Average Bacterial Sequences	Average Archaeal Sequences	ANOSIM near/away Bacteria	ANOSIM near/away Archaea
		Bacterial Shannon Diversity (rarefied)	Archaeal Shannon Diversity (rarefied)	Bacterial OTUs	Archaeal OTUs	Bacterial Shannon Diversity (rarefied)	Archaeal Shannon Diversity (rarefied)	Bacterial OTUs	Archaeal OTUs				
*Halo*	0–10	10.2 (9.4)	6.3 (5.8)	2947	1158	8.3 (7.8)	3.6 (3.4)	1871	573	8188	44473	0.26 (p < 0.05)	0.06 (p < 0.05)
	11–22	10.0 (9.2)	6.5 (6.1)	2529	1106	9.5 (8.9)	6.8 (6.7)	1895	974	8544	39915		
Ewing Bank	0–10	9.9 (9.2)	5.6 (5.3)	2315	589	7.2 (7.1)	3.4 (3.3)	894	133	5459	20794	0.33 (p < 0.001)	0.31 (p < 0.05)
	11–22	9.8 (9.1)	6.5 (6.1)	2151	682	9.6 (9.0)	5.9 (5.4)	2050	683	9188	30338		
Mica	0–10	9.5 (8.9)	4.7 (4.4)	1944	530	8.0 (7.5)	3.7(3.6)	1615	332	6414	35222	0.11 (p = 0.2)	0.02 (p = 0.49)
	11–22	9.8 (9.1)	5.7 (5.4)	2049	697	9.9 (9.0)	6.4 (6.2)	2329	932	8153	38228		
*U*-166	0–10	9.1 (8.6)	4.4 (4.2)	1623	353	7.2 (7.0)	4.1 (4.1)	898	352	5072	37701	0.03 (p = 0.28)	0.04 (p = 0.24)
	11–22	9.3 (8.9)	6.5 (6.2)	1844	590	9.4 (8.9)	6.1 (6.3)	1790	631	7594	32313		
Mardi Gras	0–10	6.9 (6.8)	3.3 (3.2)	709	140	7.6 (7.3)	4.2 (4.1)	1236	196	3451	13816	0.14 (p = 0.26)	0.11 (p = 0.20)
	11–20	nd	nd	nd	nd	9.4 (8.8)	5.4 (5.9)	1912	340	8316	23694		
*Anona*	0–10	9.6 (8.9)	4.9 (4.7)	2254	530	nd	nd	nd	nd	9571	51636	nd	nd
	11–18	9.2 (8.7)	5.6 (5.4)	1621	467	nd	nd	nd	nd	6758	26194		
Viosca Knoll	0–10	7.8 (7.3)	3.6 (3.5)	1456	355	8.0 (7.5)	3.9 (3.7)	1764	496	5970	28145	0.20 (p < 0.05)	0.22 (p < 0.05)
	11–20	9.8 (9.2)	5.8 (5.4)	2143	819	9.8 (9.2)	5.8 (5.4)	2291	797	7900	40474		

nd = no data.

**Figure 3:**
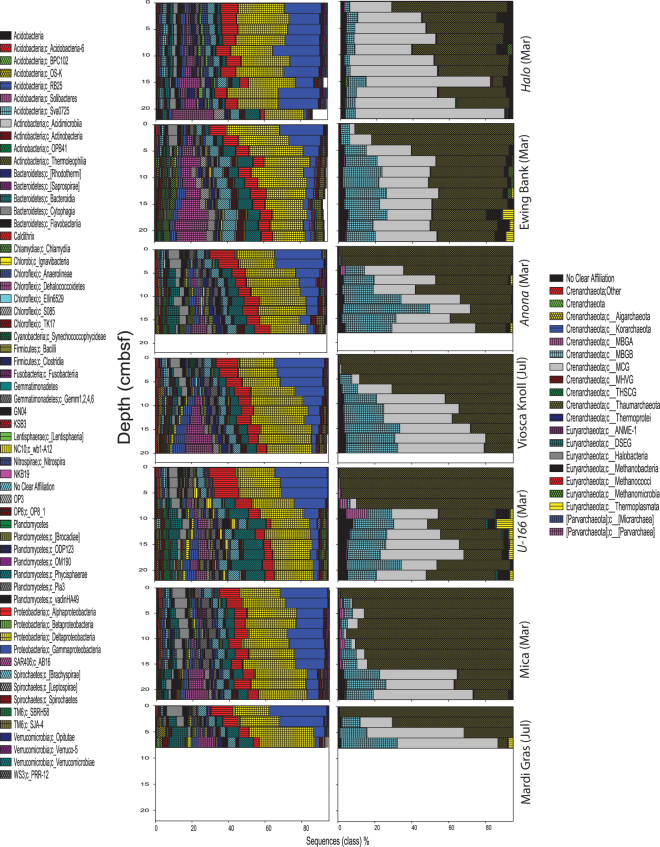
Bacterial (left) and archaeal (right) community composition in push cores samples collected within 2 m of seven shipwreck sites during PE14-15 (March 2014) and PE15-02 (July 2014). Data for each site are displayed as depth profiles in cm below seafloor (cmbsf). Class level relative sequence abundance for all major classes (representing greater than 1% of total population) are displayed. Plots created in SigmaPlot (v.13.0).

The Delta-, Gamma- and Alpha-proteobacteria—were highly abundant at all sites (Figs 3 and S1). Deltaproteobacteria averaged 24% of sequences (range 17–26%), and increased slightly with depth. Gammaproteobacteria averaged 12% of sequences, (range 0.9–33%) and were most abundant in surface sediments. The Alphaproteobacteria were third most abundant, averaging 8% of all sequences (range 2–20%), with highest abundances in surface sediments at moderately and heavily impacted sites. The Dehalococcoidetes and Phycisphaerae averaged 5% of sequences (range 0–27% and 2–27% respectively), and generally increased with depth. Sediments at reference sites had the largest percentage of Dehalococcoidetes. Sediments at *U*-166 and the Mardi Gras Wreck had the largest percentage of Phycisphaerae.

**Figure 4:**
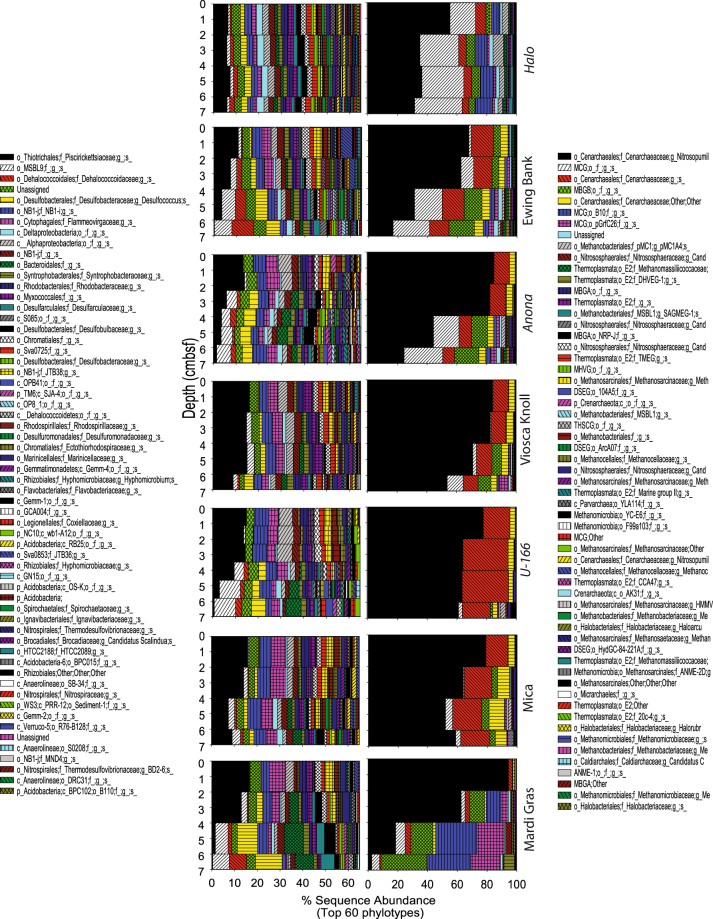
The top 60 most abundant OTUs in the sediment dataset for bacteria (left) and archaea (right). Data for each site are displayed as depth profiles down to seven cm below seafloor (cmbsf). Plots created in SigmaPlot (v.13.0).

Although Deltaproteobacteria were the most abundant class, one Gammaproteobacteria phylotype related to the Piscirickettsiaceae family accounted for up to 16% of sequences in surface sediments (0–5 cm below seafloor or cmbsf), and was uniformly abundant in the upper 4–6 cmbsf of cores collected proximate to the Viosca Knoll Wreck, *U*-166, the Mica Wreck, and the Mardi Gras Wreck (Fig. 4). The second most abundant phylotype was a member of the candidate order MSBL9 of the Phycisphaerae. Members of this candidate order may be involved in breakdown of complex polysaccharides^15^.

Sequences classified to the Dehalococcoidaceae, which are ubiquitous in marine sediments^16^, were third most abundant in the dataset. Their abundance was driven by prevalence in the lower half of cores from reference sites (Fig. 3). The most abundant Deltaproteobacteria was affiliated with the sulfate-reducing *Desulfococcus* genus. This phylotype was elevated towards the base of the core collected 2 m from the Mardi Gras Wreck, where it accounted for 12% of sequences. The uncultivated order NB1-j also contributed to the abundance of Deltaproteobacteria. NB1-j sequences averaged 6% of sequences in the upper 6 cmbsf at *U*-166 and the Mica Wreck (Fig. 4). NB1-j sequences have also been observed in high abundance in sediment and coral floc collected in the immediate vicinity of *U*-166 in December 2010^17^.

The archaea libraries were dominated by fewer OTUs, and the top 60 accounted for nearly 100% of sequences in samples (Fig. 4). The Thaumarchaeota were dominant in surface sediments at all sites, and sharply declined with depth (Figs 3 and S2). Members of this class accounted for 53% (range 0.7–99%) of all archaeal sequences. The Marine Crenarchaeal Group (MCG) was the second most abundant class (25%; range 0.1–83%). The MCG were most abundant at *Halo*, and generally increased with sediment depth. The Marine Benthic Group B (MGCB) averaged 10% of sequences (range 0–47%), and increased sharply in abundance below 5 cmbsf at most sites. The Thermoplasmata, Methanobacter, and Marine Benthic Group B classes were also well represented in the dataset, averaging 3.3%, 2.5%, and 1.2% of sequences respectively (range 0–14%, 0–17%, and 0–13%, respectively).

The majority of Thaumarchaeota were affiliated with a phylotype related to the ubiquitous^18^ marine *Nitrosopumilus*, which averaged 38% of all archaeal sequences proximate to and away from shipwrecks. Sequences of this genus were observed within the DWH subsurface plume; however, they are not associated with hydrocarbon degradation. These were uniformly abundant in the upper 6 cmbsf at the Viosca Knoll Wreck, *U*-166, and the Mica Wreck (Fig. 4), and accounted for >78% of the microbiome at the surface (0–2 cmbsf) of all moderately and heavily impacted sites. At the Mardi Gras Wreck, *Nitrosopumilus* accounted for 94% of all archaeal sequences at the sediment-water interface. An unidentified MCG was second-most abundant, averaging 17% of all sequences (range 0–45%). This phylotype was most abundant towards the base of *Halo* and Ewing Bank Wreck cores. Two additional Thaumarchaeota phylotypes, affiliated with the Cenarchaeaceae, averaged 10% and 4% of sample sequences. Neither of these could be resolved beyond the family level. The more abundant of these was found at all sites, although were minimally abundant at the Mardi Gras Wreck. There were no clear trends with depth for either phylotype.

**Figure 5:**
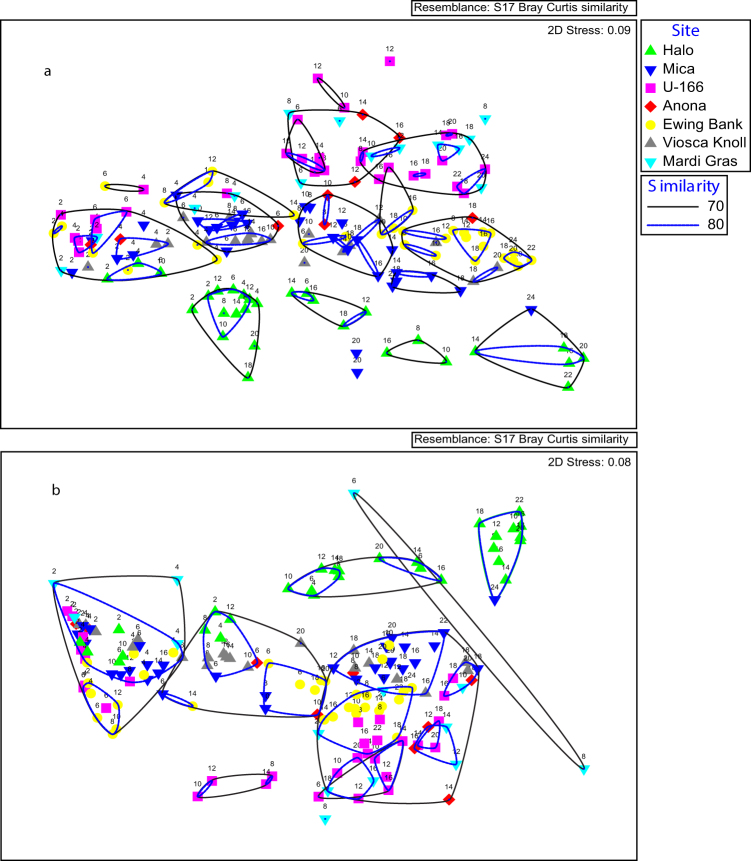
Non-metric multidimensional scaling analysis of bacterial (**a**) and archaeal (**b**) communities at GOM-SCHEMA sites. Samples included on the chart were collected proximate to (2 m) and away from shipwrecks. Hierarchical clustering was used to obtain similarity dendrograms, based on the group average linkage, which are projected on the plot as contours. The numbers on the plots correspond to sample depth in individual cores in centimeters below the seafloor (cmbsf). Plots created in Primer-E (v. 6.1.11).

Bacteria at *Halo* formed distinct clusters in non-metric multidimensional scaling (NMDS) plots of Bray-Curtis similarity data for sequence types and abundance (Fig. 5a). These sub-divided according to whether samples were collected proximate to (2 m) or away from the shipwreck (similarity profile testing, SIMPROF, p = 0.05). The majority of samples from *U*-166 and the Mardi Gras Wreck created two closely ordinated groups, regardless of proximity to the shipwrecks. Bacterial microbiomes were somewhat organized by depth. There was a cluster of samples from all sites collected at 0–5 cmbsf (Fig. 5a). Within this cluster were samples from *U*-166 down to 6 cmbsf. The similarity profile permutation test (SIMPROF) was used to identify groupings of samples at a 0.05 significance level (not shown). According to SIMPROF, these formed a statistically distinct group. Viosca Knoll Wreck, Mica Wreck, and *Anona* communities were most similar to each other, and there was a distinct cluster of 5 samples collected proximate to the Mica Wreck (Fig. 5a and SIMPROF results, not shown).

Archaea were less organized by site, with the exception of *Halo*, and more organized by depth (Fig. 5b). The NMDS included three large groups of samples: 0–8, 8–16, and 16–20+ cmbsf. At 70% similarity, there was no distinct grouping of samples according to site type (reference, moderately, or heavily impacted). However, SIMPROF revealed distinct clusters for *Halo* and the Ewing Bank Wreck (p = 0.05), and *U*-166, Mardi Gras Wreck, and *Anona*.

ANOSIM on the upper 10 cmbsf of all cores revealed that *Halo* bacterial microbiomes differed highly significantly from other shipwrecks (Table 3). This was generally the case for archaea at *Halo*. Bacterial microbiomes at the Ewing Bank Wreck and *U*-166, and bacterial and archaeal microbiomes at the Mardi Gras were distinct from all sites except *Anona*.

**Table 3:** Analysis of Similarities (ANOSIM). Analysis conducted on samples collected above 10 cmbsf *indicates significant difference (p < 0.05).

	*Halo*	Mica	*U*-166	*Anona*	Ewing Bank	Viosca Knoll	Mardi Gras
**Bacterial Phylotype Composition**							
*Halo*	0.00						
Mica	0.34**	0.00					
*U*-166	0.38**	0.16*	0.00				
*Anona*	0.36*	0.06	0.06	0.00			
Ewing Bank	0.29**	0.21**	0.12*	0.07	0.00		
Viosca Knoll	0.31*	0.10	0.22**	0.33*	0.17*	0.00	
Mardi Gras	0.49**	0.25*	0.15*	−0.01	0.21*	0.32*	0.00
**Archaeal Phylotype Composition**							
*Halo*	0.00						
Mica	0.17*	0.00					
*U*-166	0.16*	0.04	0.00				
*Anona*	0.11	0.10	0.00	0.00			
Ewing Bank	0.15*	0.04	0.04	0.08	0.00		
Viosca Knoll	0.12	−0.03	0.03	0.09	0.09	0.00	
Mardi Gras	0.24*	0.28*	0.25*	−0.10	0.25*	0.20*	0.00

**Indicates highly significant difference (p < 0.001).

The similarity percent (SIMPER) analysis was performed on square root transformed data (upper 10 cmbsf) to reveal the OTUs driving similarity between and among surface sediments at each site. The differences in bacterial microbiomes were explained by numerous OTUs (Supplementary Table S2); archaeal differences were explained by one OTU (Supplementary Table S3). Bacterial similarity within sites ranged 61–74%, with the lowest within-group similarity at the Mardi Gras Wreck and *U*-166. *Halo* and Mardi Gras Wreck bacteria had the greatest dissimilarity (Supplementary Table S2) to other sites. Dissimilarity between *Halo* and other sites was driven by Dehalococcoidaceae-related OTUs. Dissimilarity of the Mardi Gras Wreck to other sites was driven by Piscirickettsiaceae-related OTUs. Archaeal within-site similarity ranged 59–74% (Supplementary Table S3). Archaea at the Mardi Gras Wreck had the greatest dissimilarity to other sites, followed by *Halo*. In all comparisons, either the *Nitrosopumilus*-related OTU, or MCG-related OTUs explained 11% or more of the dissimilarity between sites.

Phylogenetic Investigation of Communities by Reconstruction of Unobserved States (PICRUSt)^19^ was used to predict metagenomes of bacterial taxa. The analysis was not conducted on archaea given a poor return of OTUs in closed reference OTU picking. Predicted functional genes involved in PAH and naphthalene degradation and metabolism of xenobiotics by cytochrome P450 were elevated in surface sediments at *Anona*, *U*-166 and the Mardi Gras Wreck (Supplementary Fig. S3).

### Sediment Physical Properties

Cores from *U*-166 had a reddish-brown layer at the surface that extended down to ~5 cmbsf. The layer was unconsolidated, gelatinous, and stained nitrile gloves in a manner consistent with observations of MOSSFA made by others (Joye *et al*.^20^ and Ziervogel *et al*.^21^) in cores from the Mississippi Canyon leasing area.

**Figure 6:**
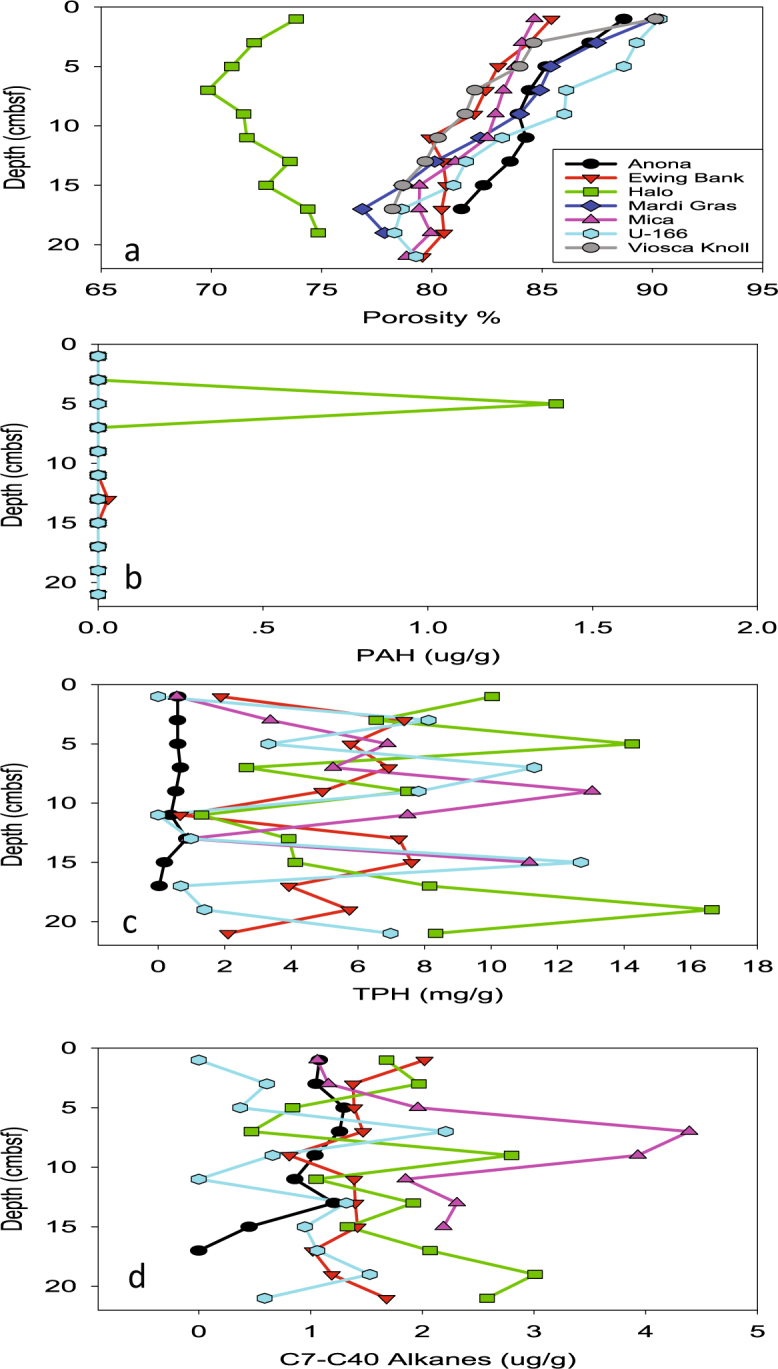
Sediment porosity, PAH, TPH, and long-chain n-alkanes (C7–C40) in sediment samples collected proximate to shipwrecks during PE14-15. Mardi Gras and Viosca Knoll porosity samples were collected during PE15-02. No hydrocarbon data are available for those sites. Plots created in SigmaPlot (v.13.0).

Highest porosities were observed at the surface at Mardi Gras and Viosca Knoll Wrecks, and *U*-166 (Fig. 6a). At the former two, porosity decreased through the upper 6 cmbsf, while at *U*-166 it remained high (~90%). Porosity at *Halo* was significantly reduced compared to other sites. This relatively shallow site (Table 1) experiences high current velocities as was observed during both dives in 2014, along with poor visibility (~3 m). As a result, sediments at *Halo* are sandy with larger grain size and lower water content.

### Hydrocarbon analysis

PAH concentrations were near the limit of detection (LOD) in most cores, likely because samples were collected four years post-spill. The low concentrations could have further been driven by potential sorption of PAHs to whirl-pak bags used for sample collection (Fig. 6b). However, a peak at 6 cmbsf at *Halo* was detected. Total petroleum hydrocarbons (TPHs) ranged 0–17 mg/g, and were elevated at *Halo*, *U*-166, and Mica Wreck cores various depths from surface to bottom (Fig. 6c). *Halo*, an oil tanker, was carrying crude oil when it sank in 1942, which explains observations in this core. *U*-166 and the Mica Wreck had peak TPH concentrations at mid-core. PAHs and TPHs were near the LOD at *Anona*. The concentration of C7-C40 alkanes ranged 0–4 μg/g in all samples (Fig. 6d). Peak concentrations of C7-C40 alkanes were observed mid-core at the Mica Wreck, *U*-166, and *Halo*, the latter having a secondary peak at the base of the core.

Sediment radiocarbon natural abundance (Δ^14^C) was used to indicate if petrocarbon (presumably oil) was deposited near shipwrecks (Table 4). The analysis assumes that surface sediment contains a more modern Δ^14^C signature than ‘fossil’ hydrocarbons^4^. The approach is powerful when tracking the fate of weathered oil (~petrocarbon) through the Gulf ecosystem^4^. *Halo* surface and deep sediments were significantly Δ^14^C depleted (Table 4). Sediments at 18–20 cmbsf collected ~2 m from *Halo*’*s* hull near a breach point created by the 1942 torpedo strike have a nearly fossil petroleum Δ^14^C signature (−1000‰). This signal is most likely attributable to the crude oil that the vessel was carrying when it sank. In all sites other than *U*-166, age increased with depth. At *U*-166, there was no change in Δ^14^C with depth.

**Table 4:** Sediment radiocarbon age analysis on discrete samples collected during the April 2014 cruise, and ^210^Pb analysis on cores collected during the July 2014 cruise to determine sedimentation rate.

Site	Depth (cmbsf)	d ^13^C	^14^C age, years BP	D^14^C	0–10 cmbsf Sedimentation Rate (cm/year)
*Halo*	0–2	−24	7340	−598.9	0.08
	18–20	−27.8	28660	−971.8	
Mica	0–2	−21.7	1830	−204.1	0.14
	18–20	−21.1	3160	−325.4	
*U*-166	0–2	−22.1	2590	−275.9	0.63
	18–20	−21.3	2540	−271.5	
Mardi Gras	0–10	nd	nd	nd	0.05
*Anona*	0–2	−21.1	1270	−146.4	0.26
	16–18	−20.6	2230	−242.7	
Ewing Bank	0–2	−22.4	2330	−251.6	0.12
	18–20	−22	3630	−363.4	

The rate of sedimentation was analyzed at *Halo*, the Mica Wreck, *U*-166, the Mardi Gras Wreck, *Anona*, and the Ewing Bank Wreck (Table 4 and Supplementary Fig. S4). Through the upper 10 cmbsf, the rate ranged from 0.08 to 0.63 cm year^−1^. The lowest rates were observed at reference sites *Halo* and the Ewing Bank Wreck, and the highest at *U*-166. The Pb-210_xs_ activity profiles (Supplementary Fig. S4) from *Halo*, the Mica Wreck, *Anona*, and the Ewing Bank Wreck displayed expected sediment accumulation profiles with depth. However, the Pb-210_xs_ profile for *U*-166 was anomalous relative to other sites. Specifically, the upper 5 cm of the *U*-166 core indicate that old sediment was deposited over younger surficial sediments, while the underlying sediment from 6–10 cmbsf displays a normal accumulation profile, similar what was observed at the Mica Wreck and Mardi Gras Wreck profiles. The full profile at *U*-166 determined an anomalously high sedimentation rate of 0.63 cm year^−1^ through 10 cmbsf.

## Discussion

Microbiome, physical property, and chemical data comparisons were used to evaluate the impact of the DWH spill to sediments surrounding historic shipwrecks. To rule out the influence of sediment berms formation around these high-relief structure, or the physical disruption of sediments during wrecking events, analyses were conducted in proximity to, and away from each shipwreck’s lateral extent.

At *Halo* and the Ewing Bank and Viosca Knoll Wrecks, bacterial and archaeal microbiomes were significantly different proximate to vs. away from the shipwrecks. This was not the case at the heavily impacted sites (Mica Wreck, *U*-166, and Mardi Gras Wreck). Shannon diversity was generally higher in surface sediments near the shipwrecks as compared to away from them, with the exception of the Mardi Gras Wreck. Shipwrecks are described as islands of biodiversity owing to the higher alpha diversity of macrobiota on shipwrecks compared to the surrounding environment. However, shipwreck microbiomes have never been evaluated. This study is the first to evaluate the effect of shipwreck proximity on microbiome diversity in deep-sea sediments, albeit with coarse resolution. The results of the ANOSIM and alpha diversity analyses indicate that an island effect may be imparted on some sediment microbiomes as a result of proximity to shipwrecks. However, at the spill impacted sites Mica, *U*-166 and Mardi Gras, this effect was not evident, either due to the greater depth of these sites, or possibly the presence of spill residues.

The area that *U*-166 and the Mardi Gras Wreck are located in has been intensely sampled from 2010 to 2014^1,6^. Impacts to sediment and coral microbiomes in this location have been reported by other research groups^8,17,22,23^. Our study found similarities with these prior works. First, domination of surface sediments by unidentified sequences associated with the Thiotrichales, specifically, the Piscirickettsiaceae, was observed. Two studies of DWH spill-impacted benthic^17^ and pelagic communities^18^ noted significant enrichment of Thiotrichales. At *U*-166 and the Mardi Gras Wreck, Piscirickettsiaceae-related sequences accounted for 12–16% of all sequences in surface sediments. At these locations, Gammaproteobacteria, commonly observed in deep marine sediment, averaged 30% of bacterial sequences at the surface of cores nearest the shipwrecks. Yang *et al*.^23^ observed Gammaproteobacteria to average 23% of sequences in surface sediments prior to the arrival of DWH spill contaminants on the seafloor within 4 km of the Macondo well. In Yang’s study, the percent of Gammaproteobacteria rose to 33%, post-spill in line with observations in the present study. This finding suggests that the spill impact on surface sediments was still evident four years later. The PICRUSt prediction data also indicate an increase in KEGG pathways involved in PAH metabolism in bacterial communities suggesting impacts on function at sites within the spill’s footprint.

The most abundant Deltaproteobacteria at the Mardi Gras Wreck was affiliated with the sulfate-reducing *Desulfococcus*. Members of this genus were among the top-ranked phylotypes in metagenomes created for samples less than 3 km from the wellhead in 2010^22^, and Desulfobacteraceae were also elevated in 16S rDNA profiles of spill-contaminated sediment samples in the vicinity of the Mardi Gras Wreck in October 2010^23^. *Desulfococcus* sequences and sequences affiliated with the NB1-j were also in elevated at sites collected near the wellhead in this study and a previous work^17^.

Prior studies have addressed water column archaea in context with the DWH spill and have noted high abundances of Thaumarchaeota^24,25^. These prior works showed that their presence and abundance was consistent in samples collected within and outside of the DWH subsurface plume; accordingly, they are thought to have no role in hydrocarbon degradation. *Nitrosopumilus maritimus*-affiliated sequences in those studies were only slightly depressed when amended with oil in laboratory experiments, further suggesting that spill-related contaminants may have no significant impact on these water column archaea. However, Yergeau^26^ noted enrichment of Thaumarchaeota affiliated with the *Nitrosopumilus* genus in deep-water samples located within the subsurface plume. *Nitrosopumilus*-related sequences were the most abundant archaea at all sites in the current study, suggesting that they are a poor marker for spill contamination in sediments. However, we note their significant enrichment in surface sediments at the Mardi Gras Wreck relative to other locations.

**Figure 7:**
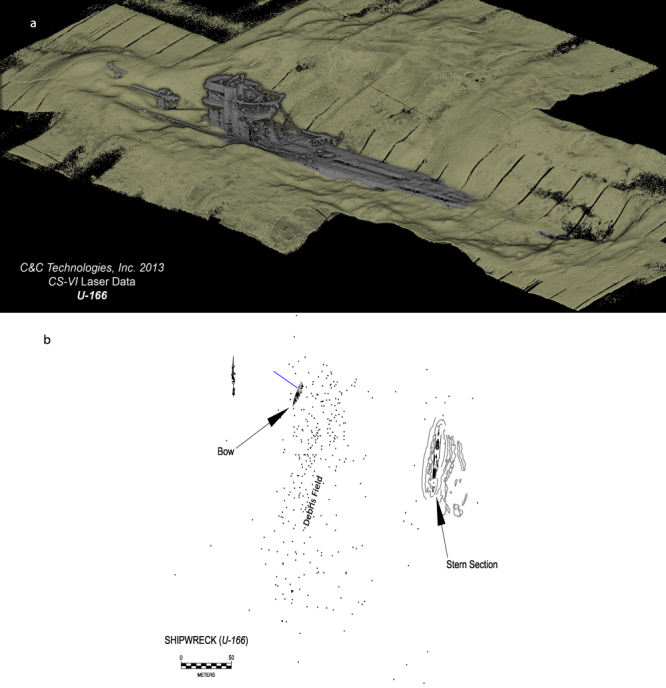
Perspective of 3D laser scanned data collected at the *U*-166 stern section depicting sediment berms around the hull and impact crater (**a**). 2013 imagery by C&C Technologies, Inc. (now Oceaneering Inc.). Site diagram of *U*-166 depicting the stern and bow section, and location of sampling effort for this study (**b**).

The physical property data and geochemical data provide evidence that *U*-166 and the Mardi Gras Wreck experienced hydrocarbon contamination as a result of the DWH spill. These sites had surface porosities of ~90%, which exceeds other locations in this study, and average porosities for Mississippi Canyon^1^. Ziervogel and colleagues noted elevated sediment porosity with greater proximity to the Macondo well in their study^21^. Porosity can be altered through physical manipulation of sediment, or through deposition of highly porous (96–99%) MOSSFA^5^. Surface porosities in this study at *U*-166 and the Mardi Gras Wreck were elevated proximate to and away from these shipwrecks indicating that lower surface porosity cannot be attributed to the wrecking events. This was important to delineate at *U*-166 as the wrecking event resulted in a 3-m impact crater that the main wreckage rests in (Fig. 7a). Notably, samples for this study were not collected near the main *U*-166 wreckage to avoid what is ostensibly a sediment trap, and instead samples were collected at the bow section disarticulated from the main wreckage, ~150 m to the northwest (Fig. 7b).

The PAH data for this study are inconclusive in terms of linking the observed hydrocarbons to residual DWH spill contaminants. TPH and long-chain n-alkane data offer evidence of contamination at *U*-166, but were unavailable for the Mardi Gras Wreck. However, the radiocarbon and Pb-210_xs_ activity data for this study provide compelling evidence for a sedimentation event at both locations. Riverine organic matter (OM) Δ^14^C ranges from −86 to −223‰^4^. Surface sediments in Mississippi Canyon ordinarily reflect the signature of new OM and are Δ^14^C enriched. With increasing depth, samples should become more Δ^14^C depleted. These are guides that must take into account location relative to the Mississippi River plume source of OM. However, the profile shape observed at *U*-166, specifically the deviation from an expected Δ^14^C enriched signature at surface and depleted signature below, is indicative of either lateral transport of aged material^27^ or a sedimentation event. Brooks *et al*.^28^ observed Δ^14^C depleted surface sediments in cores from DeSoto canyon, collected in December 2010. The Δ^14^C in the upper 1 cm of that study was nearly the same as at 15 cmbsf, however, Δ^14^C enrichment was observed through 1–5 cmbsf.

In the current study, evidence for a sedimentation event is also found in the modeled sedimentation rates provided by the Pb-210_xs_ data. A 2004 study^29^ in the Mississippi Canyon leasing area revealed that sedimentation rates were 0.09 cm/year. This rate is consistent with rates in other coastal areas at similar depths^27^, and the average for the past 100 years in DeSoto Canyon, north east of the study area^28^. Sedimentation rates at *Halo* and the Ewing Bank Wreck are consistent with prior observations^29^. A normal decay pattern for Pb-210_xs_ (decreasing with depth) was evident for all sites except *U*-166, as was Δ^14^C attenuation with depth at all sites except *U*-166. The Pb-210_xs_ profile at *U*-166 immediately declined and then spiked at 6 cmbsf, nearly equivalent to the surface value of Pb-210_xs_. A sedimentation rate at this location of 0.63 cm/year is seven times higher than expected, based on pre-spill estimates^29^. Brooks *et al*.^28^ used short-lived radioisotopes (i.e., ^7^Be and ^234^Th), to compare sediment accumulation across a time series from 2010 to 2012. The study revealed a brief but rapid depositional event in DeSoto Canyon that occurred as a consequence of MOSSFA sedimentation during and immediately following the DWH spill. Taken together with the Δ^14^C profile at *U*-166, it is evident that a similar rapid sedimentation event occurred at *U*-166, and buried younger sediments.

Previous studies support our finding of a rapid sedimentation event following the DWH spill^28^. However, sediment dynamics in the Mississippi Canyon leasing area is complicated by lateral shelf-slope transport and seafloor currents. Shipboard Acoustic Doppler Current Profiler was only able to resolve currents to ~700 m, and thus, no data is available for *U*-166. Seafloor currents can be disrupted by high-relief structures, although, sampling occurred where no berm formation was evident. Currents below 1000 m can exceed critical shear stress (0.1 to 0.4 Pa) that would induce incipient motion and facilitate lateral transport^30,31^. However, vane sheer strength at *U*-166 ranged from 0.42 to 0.65 Pa in the upper 6 cmbsf, proximate to and away from the shipwrecks, thus, lateral transport of sediment would not provide sufficient sediment entrainment to account for what amounts to massive deposits (~5 to 6 cm) at *U*-166.

## Conclusions

This study is the first to examine sediment microbiomes proximate to deep-sea historic shipwrecks. The purpose of this was to reveal if historic shipwrecks were impacted by the DWH spill. Close to the Macondo wellhead, there is evidence for oiling of *U*-166—a historically significant shipwreck, war grave, and ecological resource. Sedimentation rates at *U*-166 cannot be attributed to riverine sediment load or ordinary resuspension and lateral transport of seafloor sediments. The *U*-166 Pb-210_xs_ profile displayed a 5-cm layer of older sediment overtop younger material. The porosity of that interval, and Piscirickettsiaceae sequence abundance are both uniform and high. Radiocarbon data reveals surface sediments with the same approximate age as at the base of the core. It is likely that the Mardi Gras Wreck also experienced fallout of DWH contaminants, given its location and the commonalities between its microbiome and that of *U*-166. However, due to a lack of hydrocarbon data for the site, and a Pb-210_xs_ profile that indicates markedly lower sedimentation rates than at *U*-166, this cannot be definitively stated. The Mica Wreck also bears high microbiome similarity with *U*-166 and the Mardi Gras Wreck, but deviates on physical and chemical parameters. The Mica Wreck is located north of the fallout plume, and possibly narrowly avoided the DWH sedimentation event. Prior studies have identified a patchy distribution of impacts from the spill on benthic habitats and microbiomes in the area around the wellhead^1,2,6,7^.

The study also examined the influence of shipwrecks on microbiome diversity in sediments surrounding anthropogenic structures. Shipwreck proximity positively influenced diversity in surface sediments near World War II-era and 19^th^ century shipwrecks. Shipwreck-associated microbiomes were distinct from the surrounding environment, at four of 7 locations, indicating that some shipwrecks impart an island effect on seafloor microbiomes. However, at the Mica and Mardi Gras Wrecks, and *U*-166, diversity and composition were similar near and away from the sites. This may suggest the effect is not present at these deeper sites, or that spill associated effects obscure evidence for an island effect.

## Materials and Methods

### Sample Collection

Sediment was collected during two expeditions: PE14–15 and PE15–02 in March and July 2014, respectively, as part of the Gulf of Mexico Shipwreck Corrosion, Hydrocarbon Exposure, Microbiology, and Archaeology (GOM-SCHEMA) project (https://www.boem.gov/GOM-SCHEMA) using the *Global Explorer* remotely operated vehicle (GEX-ROV - Deep Sea Systems International, http://www.deepseasystems.com). Prior to sample collection, a visual survey of each shipwreck was performed to update archaeological site plans and identify suitable areas for sediment sampling that are devoid of archaeological materials.

Push core samples were obtained using a 7-function manipulator arm without damaging the archaeological sites or associated debris fields within 2 m of each shipwreck, and 100–200 m away from each shipwreck (Fig. 2). Replicate cores for various analyses were obtained and sampled according to standard techniques (See Supplementary Materials).

### DNA extraction and sequencing of 16S rRNA gene amplicons

Genomic DNA was extracted using a modification of the FastDNA™ protocol described in a previous study^32^ and 16S rRNA gene amplification and sequencing was carried out according to Comeau *et al*.^33^ at the Microbiome Resource (IMR) facility at Dalhousie University. Bioinformatics, sequence quality control, and calculation of alpha diversity statistics (Shannon Diversity, Good’s Coverage) were carried out in a pipeline generated with UPARSE^34^ and Quantitative Insights into Microbial Ecology (QIIME)^35^ (See Supplementary Materials). Shannon Diversity was calculated for both rarefied and un-rarefied datasets. Data were also processed through PICRUSt 1.1.0 to predict metagenome functional content^19^.

### Sediment Physical Properties

Sediment porosity was determined from wet and dry weights at 1 cm intervals. Grain density was determined using a PentaPycnometer. Sediment accumulation rates were determined using Pb-210_xs_ activity measurements of sediments from the top 10 cmbsf at the Louisiana State University, Department of Geology.

### Hydrocarbon Analysis

Analyses were not conducted on Mardi Gras and Viosca Knoll samples, as they were not visited during the first GOM-SCHEMA cruise in March 2014 due to weather complications. Samples were analyzed at the University of Georgia (UGA) with a protocol developed by Yi Yang (See Supplementary Materials). Sediments were sequentially extracted with dichloromethane:methanol (9:1, vol/vol) and hexane in a horn ultrasonicator. Combined extracts were reduced using a Rotovap^®^, desulfurized, washed, and further reduced under nitrogen stream prior to analysis. Hydrocarbons were analyzed using a LECO Pegasus 4D GCxGC-Time of Flight Mass Spectrometer system equipped with an electron ionization source. PAH (TCL PAH mix, Supelco, Bellefonte, PA) and TPHs (BP surrogate oil) standards were used as references to quantify PAHs and TPHs concentrations in extracts.

### Radiocarbon Analysis

Samples were analyzed for radiocarbon natural abundance at the Center for Applied Isotope Studies (CAIS) at UGA according to Chanton *et al*.^4^. The delta notation (∆^14^C) is used to report the measured isotope distributions.

### Statistical analyses

QIIME and PRIMER v. 6.1.13 were used for statistical analysis of microbiomes^36–38^. Bray-Curtis dissimilarities were calculated from sequence abundance. NMDS was performed to yield a ‘best fit’ 2D graphical representation of similarities in sample microbiomes. Hierarchical clustering (CLUSTER) was used to generate similarity dendrograms based on group average linkage. ANOSIM was used to identify differences in microbiomes between sample groups. SIMPER with a 90% cutoff ranked the percent contribution of phylotypes to within group similarity or between group differences.

### Data availability

Sequences from this study are published under Bioproject number PRJNA401282. All data generated or analyzed during this study are in this published article and its Supplementary Information). The locations of shipwrecks are not publically available.

## Electronic supplementary material

Supplementary Information
