# Supplementary material for: The impact of the Deepwater Horizon blowout on historic shipwreck-associated sediment microbiomes in the northern Gulf of Mexico
Source: Sci Rep. 2018 Jun 28;8:9057. doi: 10.1038/s41598-018-27350-z (PMC6023898; doi:10.1038/s41598-018-27350-z)
Supplement: Supplementary file 1 — Supplementary Information [file 41598_2018_27350_MOESM1_ESM.pdf]

Supplementary Information

The impact of the *Deepwater Horizon* blowout on historic shipwreck-associated sediment microbiomes in the northern Gulf of Mexico

Leila J. Hamdan[1*], Jennifer L. Salerno[2], Allen Reed[3], Samantha B. Joye[4], and Melanie Damour[5]

[1]University of Southern Mississippi, Ocean Springs, MS 39564

[2]George Mason University, Manassas, VA 20110

[3] U.S. Naval Research Laboratory, Stennis Space Center, MS, 39529

[4]University of Georgia, Athens, GA, 30602

[5]Bureau of Ocean Energy Management, New Orleans, LA, 70123

*Corresponding author: Leila J. Hamdan, University of Southern Mississippi, School of Ocean Science and Technology, Division of Coastal Sciences, Ocean Springs, MS, USA. e-mail: leila.hamdan@usm.edu. Tel: (228) 818-8011

Materials and Methods

Sample Collection

Sediment was collected during two expeditions on board R/V *Pelican*: PE14-15 and PE15-02 in March and July 2014, respectively, as part of the Gulf of Mexico Shipwreck Corrosion, Hydrocarbon Exposure, Microbiology, and Archaeology (GOM-SCHEMA) project (https://www.boem.gov/GOM-SCHEMA) using the *Global Explorer* remotely operated vehicle (GEX-ROV - Deep Sea Systems International, http://www.deepseasystems.com). Prior to sample collection, a visual survey of each shipwreck was performed to update archaeological site plans and identify suitable areas for sediment sampling that are devoid of archaeological materials. During surveys, no visual evidence of shipwreck damage from NRDA or other sampling efforts was observed.

A requirement for sampling was the ability to obtain sediment within 2 m of each shipwreck without damaging the archaeological sites, including cultural materials within their associated debris fields. Using an Orion 7-function manipulator arm, *Jason*-style push corers obtained 2.5 in. inner diameter (ID) sediment cores within 2 m of each wreck, and away from the primary wreck sites, and debris fields, (100–200 m distance) (Figure 2). Project archaeologists and archaeological site plans were consulted to determine sampling locations. Replicate cores for various analyses were obtained. Cores were extruded into 2.5 in. ID tubes and sampled destructively at 2 cm resolution. Sediment for genetic analysis was placed into sterile, PCR clean tubes, immediately frozen at -20˚C, and stored at -80˚C. Samples for gravimetric porosity were collected in whirl-pack bags and stored refrigerated. Samples for hydrocarbon analysis and bulk organic carbon analysis were placed in whirl-pack bags and stored at -20˚C.

DNA extraction and sequencing of 16S rRNA gene amplicons

Genomic DNA was extracted using a modification of the FastDNA™ protocol described elsewhere [1]. Total genomic DNA was quantified using the Qubit® 2.0 Flurometric Quantitation system (Invitrogen, Carlsbad, CA, USA). Approximately 2 ng/µL of template was sent to the Integrated Microbiome Resource (IMR) facility at Dalhousie University (Halifax, Nova Scotia, Canada) for 16S rRNA gene amplification and sequencing. PCR amplifications were carried out in 50-µL reactions described by Comeau et al. [2], using fusion primers B969F/BA1406 and A956F/A1401R to target the V6-V8 regions of the 16S rRNA gene for bacteria and archaea, respectively. PCR products were screened with an Invitrogen 96-well E-gel, and duplicates were pooled into a single plate, cleaned, and normalized. Samples were analyzed on an Illumina MiSeq platform to generate paired-end sequences.

Bioinformatics, sequence quality control and calculation of alpha diversity metrics were carried out in a pipeline generated with UPARSE [3] and Quantitative Insights into Microbial Ecology (QIIME) [4]. Shannon Diversity was calculated for both rarefied and un-rarefied datasets. UPARSE was used to merge paired-end sequences with a minimum merge length of 200 base pairs (bps) and a maximum difference of 32 bps in the overlap region (20% error rate). Merged sequences were quality filtered, discarding reads with a maximum number of expected errors >0.5 (based on Phred quality scores) [5]. Sequences were dereplicated, with unique sequences serving as OTUs; remaining sequences were assigned to OTUs based on greedy clustering and ≥97% sequence similarity. Chimeric sequences were removed and singletons were included in the library. A table containing the relative abundance of OTUs in each sample was generated and used for taxonomic identification and phylogenetic alignment using UCLUST and the GreenGenes reference database (v 13.8) [6].

Bacterial 16S rRNA sequence data were also processed through Phylogenetic Investigation of Communities by Reconstruction of Unobserved States (PICRUSt 1.1.0) to predict metagenome functional content [7]. Sequences processed up to the dereplication step were run through the "pick closed reference" pipeline in QIIME. The OTU table was normalized by predicted 16S rRNA copy and through PICRUSt to predict trait abundances based on KEGG orthologs. KEGG predictions were collapsed into KEGG pathways (K3 level) and visualized using Statistical Analysis of Metagenomic Profiles (STAMP v2.1.3) [8].

Sediment Physical Properties

Sediment porosity was determined from the volume of the porewater and total volume as determined from wet and dry weights, and water and grain density. A PentaPycnometer, which uses a helium gas to fill a chamber of known size, was used to determine sediment volume and density. Sediment accumulation rates were determined using measurements of Pb-210 excess activity (Pb-210_{xs}) at the Louisiana State University, Department of Geology. The top 10 cm below the seafloor (cmbsf) were evaluated. The sedimentation rate (S) was determined as follows: $S = \frac{\lambda(z)}{\ln\frac{A_o}{A_z}}$, where *l* is the decay constant for radionuclide Pb-210 ($\ln 2/t_{1/2} = 0.03114\ yr^{-1}$), which has a half-life ($t_{1/2}$) of 22 years, *z* is the distance below the sediment from which *Ao* was determined, *Ao* is the excess activity of the radionuclide (Pb-210) in the sediment at the top interval, and *Az* is the excess activity of Pb-210 at a distance *z* below *Ao*. In the case of Pb-210, Pb-210_{xs} is measured as the total activity of Pb-210 (Pb-210_{tot}) above that which is supported by activity from other related radionuclides.

Hydrocarbon Analyses

Analyses were not conducted on Mardi Gras and Viosca Knoll samples, as they were not visited during the first GOM-SCHEMA cruise in March 2014 due to weather complications. All

glassware was washed, wrapped in foil, and combusted at 500°C for 4 hours to remove any lingering organic carbon. All non-combustible items, including PTFE stopcocks, metal tools, caps, syringes, and volumetric glassware, were all triple rinsed subsequently with methanol, DCM, and hexane, and allowed to dry before handling. Sediment samples were thawed over a 2–3-hour window at room temperature. Roughly 5–6 grams of thawed sediment was transferred into a 50 mL freshly combusted glass centrifuge tubes. The same amount of combusted sodium sulfate (i.e., 5–6 gram) was added to remove water in the sample. Then, sediment samples were extracted sequentially with two rounds of dichloromethane (DCM):methanol (9:1, 20 mL) and one round of hexane (20 mL). For each round of extraction, the sediment sample was placed in a horn ultrasonicator (XL-2000 Misonix Ultrasonic Liquid Processors, Newtown, CT, USA) for 20 minutes at the maximum setting. The 60 mL eluent from three extraction rounds were combined and reduced to about 5 mL by Buchi rotavapor (BUCHI R-210, BÜCHI Labortechnik AG, Switzerland). The reduced extracts were desulfurized by passing through an activated copper columns and then washed with 4M HCl (~ 10 mL), DI water (~ 10 mL), methanol (3 x 10 mL), DCM (3 x 10 mL), hexane (3 x 10 mL), sequentially. After desulfurization, extracts were dried by passing through columns, which were made in combusted Pasteur pipettes using a glass wool plug to retain the anhydrous sodium sulfate. The volumes of the extracts were reduced again to about 1 mL by a blowdown nitrogen flow. The extracts were transferred into the 2 mL combusted HPLC vials using 1 mL Hamilton syringe (Hamilton, Reno, NV), and the volume was recorded. The procedural blanks using combusted sand were extracted and carried through the extraction.

Hydrocarbons were analyzed using a LECO Pegasus 4D GCxGC-Time of Flight Mass Spectrometry (ToF-MS) system equipped with an electron ionization source. Briefly, the

Pegasus 4D system is comprised of a primary column (10m Rxi-5Sil, 0.25 mm ID x 0.25um df) and a secondary column (1m Rxi-17Sil, 0.15 mm ID x 0.15   m df). Leak and tune check procedures were performed daily or before sample injections to ensure leak-free and mass calibration. Perfluorotributylamine (PFTBA) was used as the mass calibration compound. All data analysis including automatic peak finding using mass spectral deconvolution were performed using the LECO ChromaTOF-HRT software (version 4.6). PAHs (TCL PAH mix, Supelco, Bellefonte, PA). Total petroleum hydrocarbons (BP surrogate oil) standards were prepared with serial diluted samples in DCM with known amounts, and used as references to quantify PAHs and total petroleum hydrocarbons concentrations in the extracts.

Radiocarbon Analysis

Prior to analysis, samples were treated with 10% HCl to remove carbonates, rinsed, freeze-dried, and ground. Samples were then analyzed for $\Delta^{14}C$. At the Center for Applied Isotope Studies (CAIS) at UGA, samples were combusted and cryogenically purified to generate clean CO_2 [9] which were then prepared as graphite targets and analyzed by accelerator mass spectrometry. The purified CO_2 gas was converted to a solid, graphitic carbon by reducing CO_2 in the presence of hydrogen (H_2) at 580ºC in an evacuated, closed system in the presence of an iron catalyst. Iron also serves as thermal conductor and binder. Water produced during the reaction was absorbed by magnesium perchlorate. The delta notation ($\Delta^{14}C$) is used to report the measured isotope distributions, as is normalizes the radiocarbon content to a $\delta^{13}C$ value of −25‰ and accounts for the time of collection. Samples generally contained ~1000 µg of carbon and the reproducibility was roughly 5%.

Statistical analyses

PRIMER v. 6.1.13 (PRIMER-E Ltd.) was used for statistical analysis of microbiome structure [10-12]. Bray-Curtis dissimilarities were calculated from sequence abundance data without transformation. Non-metric multidimensional scaling (NMDS) ordination of Bray-Curtis dissimilarities was performed to yield a 'best fit' 2D graphical representation of similarities in sample microbiomes. Hierarchical clustering analysis (CLUSTER) was used to generate similarity dendrograms based on group average linkage. ANOSIM was used to identify differences in microbiomes between sample groups. SIMPER was run with a 90% cutoff and used to rank the percent contribution of individual phylotypes to within group similarity or between group differences.

References

1 Hamdan, L. J. *et al.* Ocean currents shape the microbiome of Arctic marine sediments. *Isme Journal* **7**, 685-696, doi:10.1038/ismej.2012.143 (2013).

2 Comeau, A. M., Li, W. K., Tremblay, J. E., Carmack, E. C. & Lovejoy, C. Arctic Ocean microbial community structure before and after the 2007 record sea ice minimum. *Plos One* **6**, e27492, doi:10.1371/journal.pone.0027492 (2011).

3 Edgar, R. C. UPARSE: highly accurate OTU sequences from microbial amplicon reads. *Nat Methods* **10**, 996-998, doi:10.1038/nmeth.2604 (2013).

4 Caporaso, J. G. *et al.* QIIME allows analysis of high-throughput community sequencing data. *Nature Methods* **7**, 335-336, doi:10.1038/nmeth.f.303 (2010).

5 Edgar, R. C. & Flyvbjerg, H. Error filtering, pair assembly and error correction for next-generation sequencing reads. *Bioinformatics* **31**, 3476-3482, doi:10.1093/bioinformatics/btv401 (2015).

6 DeSantis, T. Z. *et al.* Greengenes, a chimera-checked 16S rRNA gene database and workbench compatible with ARB. *Applied and Environmental Microbiology* **72**, 5069-5072, doi:10.1128/Aem.03006-05 (2006).

7 Langille, M. G. *et al.* Predictive functional profiling of microbial communities using 16S rRNA marker gene sequences. *Nat Biotechnol* **31**, 814-821, doi:10.1038/nbt.2676 (2013).

8 Parks, D. H., Tyson, G. W., Hugenholtz, P. & Beiko, R. G. STAMP: statistical analysis of taxonomic and functional profiles. *Bioinformatics* **30**, 3123-3124, doi:10.1093/bioinformatics/btu494 (2014).

9 Chanton, J. *et al.* Using Natural Abundance Radiocarbon To Trace the Flux of Petrocarbon to the Seafloor Following the Deepwater Horizon Oil Spill. *Environ Sci Technol* **49**, 847-854, doi:10.1021/es5046524 (2015).
10 Anderson, M. J. & Walsh, D. C. I. PERMANOVA, ANOSIM, and the Mantel test in the face of heterogeneous dispersions: What null hypothesis are you testing? *Ecological Monographs* **83**, 557-574, doi:10.1890/12-2010.1 (2013).
11 Clarke, K. R. & Warwick, R. M. *Change in marine communities: An approach to statistical analysis and interpretation, 2nd Ed., PRIMER-E Ltd., .* (Plymouth Marine Laboratory, UK., 2001).
12 Change in marine communities: An approach to statistical analysis and interpretation v. 2 (Plymouth Marine Laboratories, 2006).

Supplimentary Figure Legends

Figure S1. Comparison of bacterial community composition in push cores samples collected within 2 m of seven shipwreck sites during PE14-15 (March 2014) and PE15-02 (July 2014) and samples collected away from the wreck site during July 2014. Data for each site are displayed as depth profiles in cm below seafloor (cmbsf). Class level relative sequence abundance for all major classes (representing greater than 1% of total population) are displayed.

Figure S2. Comparison of archaeal community composition in push cores samples collected within 2 m of seven shipwreck sites during PE14-15 (March 2014) and PE15-02 (July 2014) and samples collected away from the wreck site during July 2014. Data for each site are displayed as depth profiles in cm below seafloor (cmbsf). Class level relative sequence abundance for all major classes (representing greater than 1% of total population) are displayed.

Figure S3. PICRUSt predicted metagenome contributions identifying the proportion of sequences with pathway predictions for naphthalene degradation, PAH degradation, and metabolism of xenobiotics by cytochrome P450 in surface sediments. Stars on boxes represent the average for each group (site)

Figure S4. Pb-210xs activity profiles for the upper 10 cm of sediment.

Supplementary Figures

The impact of the *Deepwater Horizon* blowout on historic shipwreck-associated sediment microbiomes in the northern Gulf of Mexico

Leila J. Hamdan[1*], Jennifer L. Salerno[2], Allen Reed[3], Samantha B. Joye[4], and Melanie Damour[5]

[1]University of Southern Mississippi, Ocean Springs, MS 39564
[2]George Mason University, Manassas, VA 20110
[3] U.S. Naval Research Laboratory, Stennis Space Center, MS, 39529
[4]University of Georgia, Athens, GA, 30602
[5]Bureau of Ocean Energy Management, New Orleans, LA, 70123

*Corresponding author: Leila J. Hamdan, University of Southern Mississippi, School of Ocean Science and Technology, Division of Coastal Sciences, Ocean Springs, MS, USA. e-mail: leila.hamdan@usm.edu. Tel: (228) 818-8011

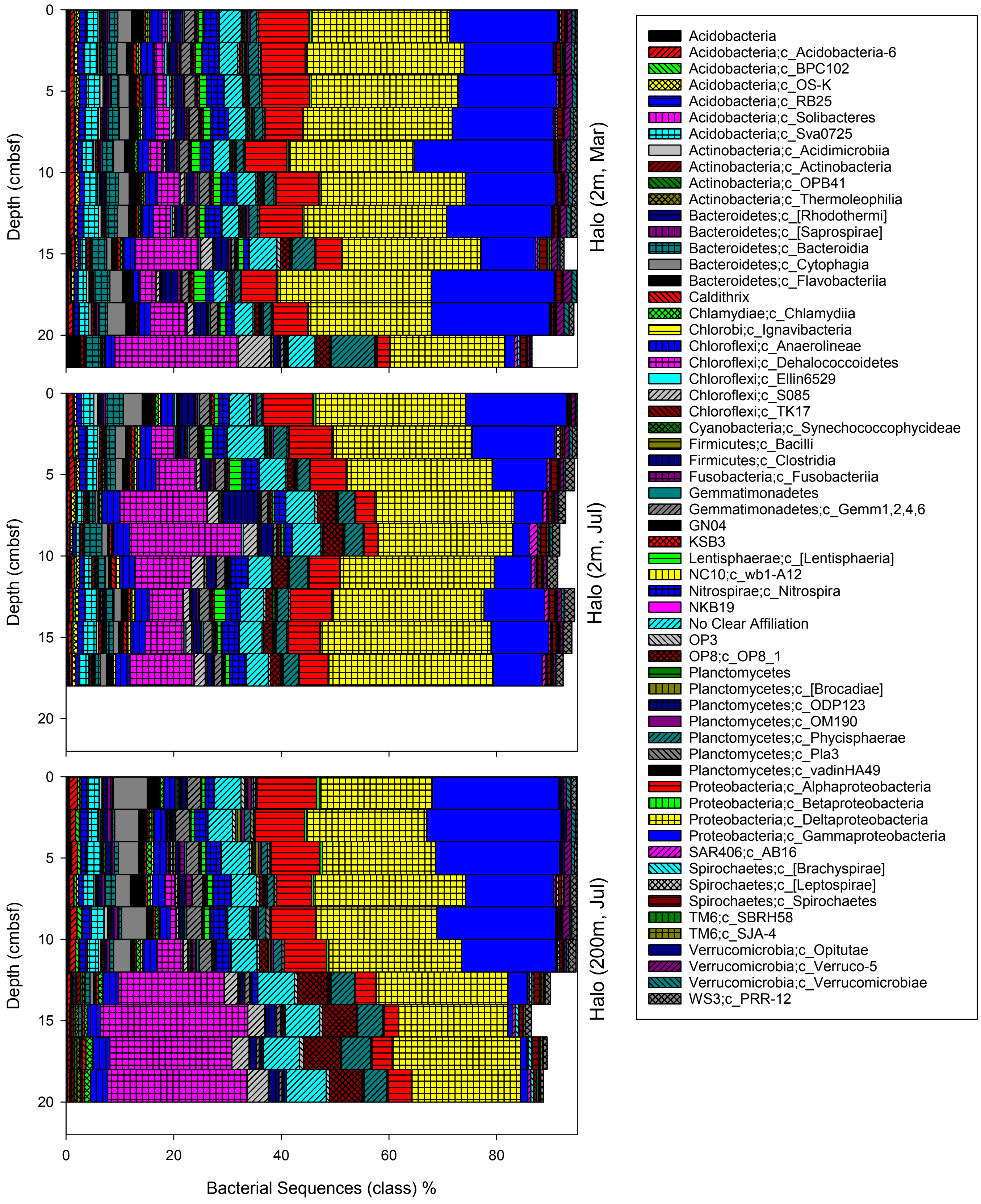
Depth (cmbsf)
0
5
10
15
20
Halo (2m, Mar)
Halo (2m, Jul)
Halo (200m, Jul)
0
20
40
60
80
Bacterial Sequences (class) %
Acidobacteria
Acidobacteria;c_Acidobacteria-6
Acidobacteria;c_BPC102
Acidobacteria;c_OS-K
Acidobacteria;c_RB25
Acidobacteria;c_Solibacteres
Acidobacteria;c_Sva0725
Actinobacteria;c_Acidimicrobiia
Actinobacteria;c_Actinobacteria
Actinobacteria;c_OPB41
Actinobacteria;c_Thermoleophilia
Bacteroidetes;c_[Rhodothermi]
Bacteroidetes;c_[Saprospirae]
Bacteroidetes;c_Bacteroidia
Bacteroidetes;c_Cytophagia
Bacteroidetes;c_Flavobacteriia
Caldithrix
Chlamydiae;c_Chlamydiia
Chlorobi;c_Ignavibacteria
Chloroflexi;c_Anaerolineae
Chloroflexi;c_Dehalococcoidetes
Chloroflexi;c_Ellin6529
Chloroflexi;c_S085
Chloroflexi;c_TK17
Cyanobacteria;c_Synechococcophycideae
Firmicutes;c_Bacilli
Firmicutes;c_Clostridia
Fusobacteria;c_Fusobacteriia
Gemmatimonadetes
Gemmatimonadetes;c_Gemm1,2,4,6
GN04
KSB3
Lentisphaerae;c_[Lentisphaeria]
NC10;c_wb1-A12
Nitrospirae;c_Nitrospira
NKB19
No Clear Affiliation
OP3
OP8;c_OP8_1
Planctomycetes
Planctomycetes;c_[Brocadiae]
Planctomycetes;c_ODP123
Planctomycetes;c_OM190
Planctomycetes;c_Phycisphaerae
Planctomycetes;c_Pla3
Planctomycetes;c_vadinHA49
Proteobacteria;c_Alphaproteobacteria
Proteobacteria;c_Betaproteobacteria
Proteobacteria;c_Deltaproteobacteria
Proteobacteria;c_Gammaproteobacteria
SAR406;c_AB16
Spirochaetes;c_[Brachyspirae]
Spirochaetes;c_[Leptospirae]
Spirochaetes;c_Spirochaetes
TM6;c_SBRH58
TM6;c_SJA-4
Verrucomicrobia;c_Opitutae
Verrucomicrobia;c_Verruco-5
Verrucomicrobia;c_Verrucomicrobiae
WS3;c_PRR-12

Figure S1.

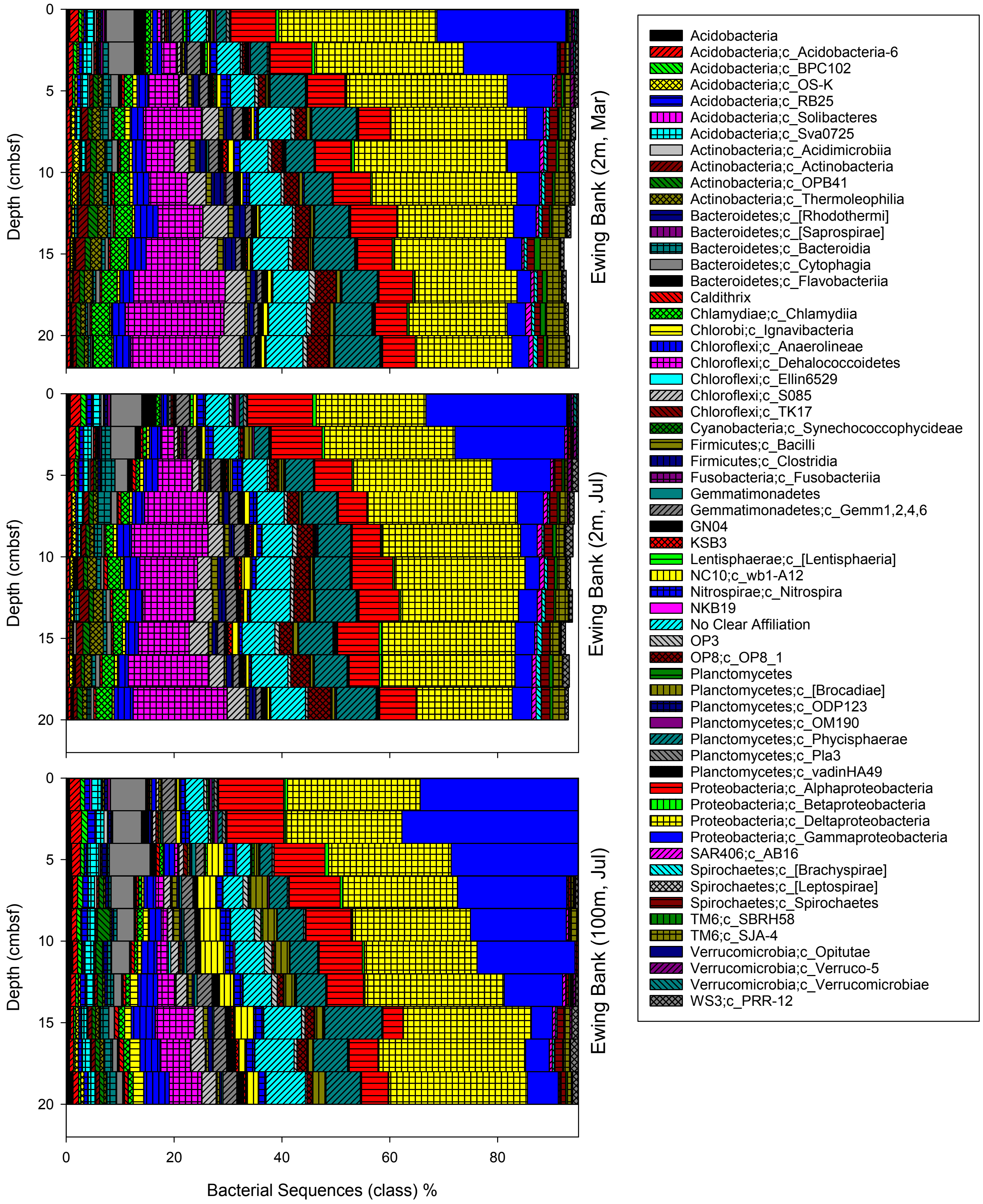

Depth (cmbsf)
Ewing Bank (2m, Mar)
Ewing Bank (2m, Jul)
Ewing Bank (100m, Jul)
Bacterial Sequences (class) %
0
5
10
15
20
40
60
80
Acidobacteria
Acidobacteria;c_Acidobacteria-6
Acidobacteria;c_BPC102
Acidobacteria;c_OS-K
Acidobacteria;c_RB25
Acidobacteria;c_Solibacteres
Acidobacteria;c_Sva0725
Actinobacteria;c_Acidimicrobiia
Actinobacteria;c_Actinobacteria
Actinobacteria;c_OPB41
Actinobacteria;c_Thermoleophilia
Bacteroidetes;c_[Rhodothermi]
Bacteroidetes;c_[Saprospirae]
Bacteroidetes;c_Bacteroidia
Bacteroidetes;c_Cytophagia
Bacteroidetes;c_Flavobacteriia
Caldithrix
Chlamydiae;c_Chlamydiia
Chlorobi;c_Ignavibacteria
Chloroflexi;c_Anaerolineae
Chloroflexi;c_Dehalococcoidetes
Chloroflexi;c_Ellin6529
Chloroflexi;c_S085
Chloroflexi;c_TK17
Cyanobacteria;c_Synechococcophycideae
Firmicutes;c_Bacilli
Firmicutes;c_Clostridia
Fusobacteria;c_Fusobacteriia
Gemmatimonadetes
Gemmatimonadetes;c_Gemm1,2,4,6
GN04
KSB3
Lentisphaerae;c_[Lentisphaeria]
NC10;c_wb1-A12
Nitrospirae;c_Nitrospira
NKB19
No Clear Affiliation
OP3
OP8;c_OP8_1
Planctomycetes
Planctomycetes;c_[Brocadiae]
Planctomycetes;c_ODP123
Planctomycetes;c_OM190
Planctomycetes;c_Phycisphaerae
Planctomycetes;c_Pla3
Planctomycetes;c_vadinHA49
Proteobacteria;c_Alphaproteobacteria
Proteobacteria;c_Betaproteobacteria
Proteobacteria;c_Deltaproteobacteria
Proteobacteria;c_Gammaproteobacteria
SAR406;c_AB16
Spirochaetes;c_[Brachyspirae]
Spirochaetes;c_[Leptospirae]
Spirochaetes;c_Spirochaetes
TM6;c_SBRH58
TM6;c_SJA-4
Verrucomicrobia;c_Opitutae
Verrucomicrobia;c_Verruco-5
Verrucomicrobia;c_Verrucomicrobiae
WS3;c_PRR-12

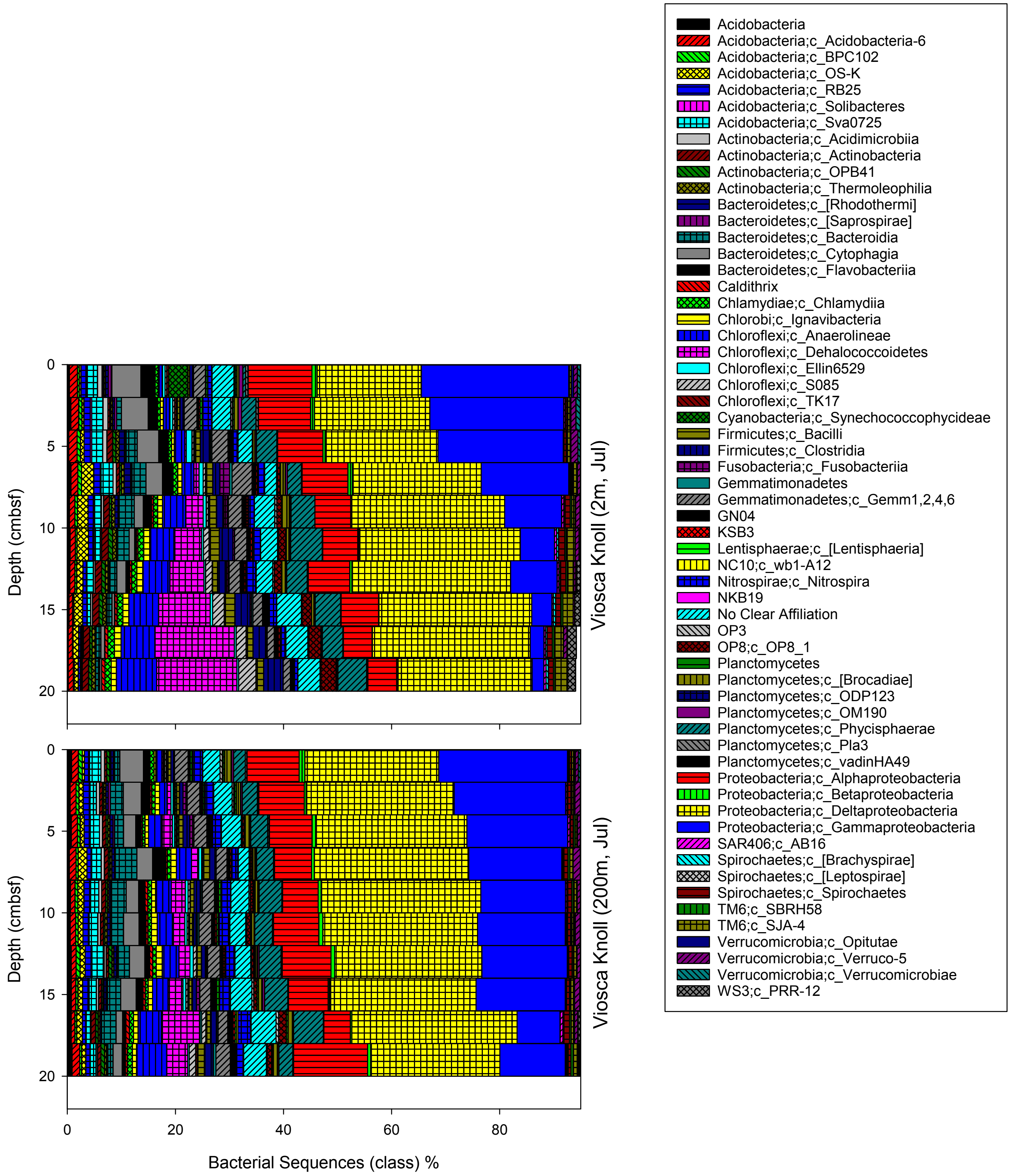

Depth (cmbsf)
Viosca Knoll (2m, Jul)
Viosca Knoll (200m, Jul)
Bacterial Sequences (class) %
0
5
10
15
20
0
20
40
60
80
Acidobacteria
Acidobacteria;c_Acidobacteria-6
Acidobacteria;c_BPC102
Acidobacteria;c_OS-K
Acidobacteria;c_RB25
Acidobacteria;c_Solibacteres
Acidobacteria;c_Sva0725
Actinobacteria;c_Acidimicrobiia
Actinobacteria;c_Actinobacteria
Actinobacteria;c_OPB41
Actinobacteria;c_Thermoleophilia
Bacteroidetes;c_[Rhodothermi]
Bacteroidetes;c_[Saprospirae]
Bacteroidetes;c_Bacteroidia
Bacteroidetes;c_Cytophagia
Bacteroidetes;c_Flavobacteriia
Caldithrix
Chlamydiae;c_Chlamydiia
Chlorobi;c_Ignavibacteria
Chloroflexi;c_Anaerolineae
Chloroflexi;c_Dehalococcoidetes
Chloroflexi;c_Ellin6529
Chloroflexi;c_S085
Chloroflexi;c_TK17
Cyanobacteria;c_Synechococcophycideae
Firmicutes;c_Bacilli
Firmicutes;c_Clostridia
Fusobacteria;c_Fusobacteriia
Gemmatimonadetes
Gemmatimonadetes;c_Gemm1,2,4,6
GN04
KSB3
Lentisphaerae;c_[Lentisphaeria]
NC10;c_wb1-A12
Nitrospirae;c_Nitrospira
NKB19
No Clear Affiliation
OP3
OP8;c_OP8_1
Planctomycetes
Planctomycetes;c_[Brocadiae]
Planctomycetes;c_ODP123
Planctomycetes;c_OM190
Planctomycetes;c_Phycisphaerae
Planctomycetes;c_Pla3
Planctomycetes;c_vadinHA49
Proteobacteria;c_Alphaproteobacteria
Proteobacteria;c_Betaproteobacteria
Proteobacteria;c_Deltaproteobacteria
Proteobacteria;c_Gammaproteobacteria
SAR406;c_AB16
Spirochaetes;c_[Brachyspirae]
Spirochaetes;c_[Leptospirae]
Spirochaetes;c_Spirochaetes
TM6;c_SBRH58
TM6;c_SJA-4
Verrucomicrobia;c_Opitutae
Verrucomicrobia;c_Verruco-5
Verrucomicrobia;c_Verrucomicrobiae
WS3;c_PRR-12

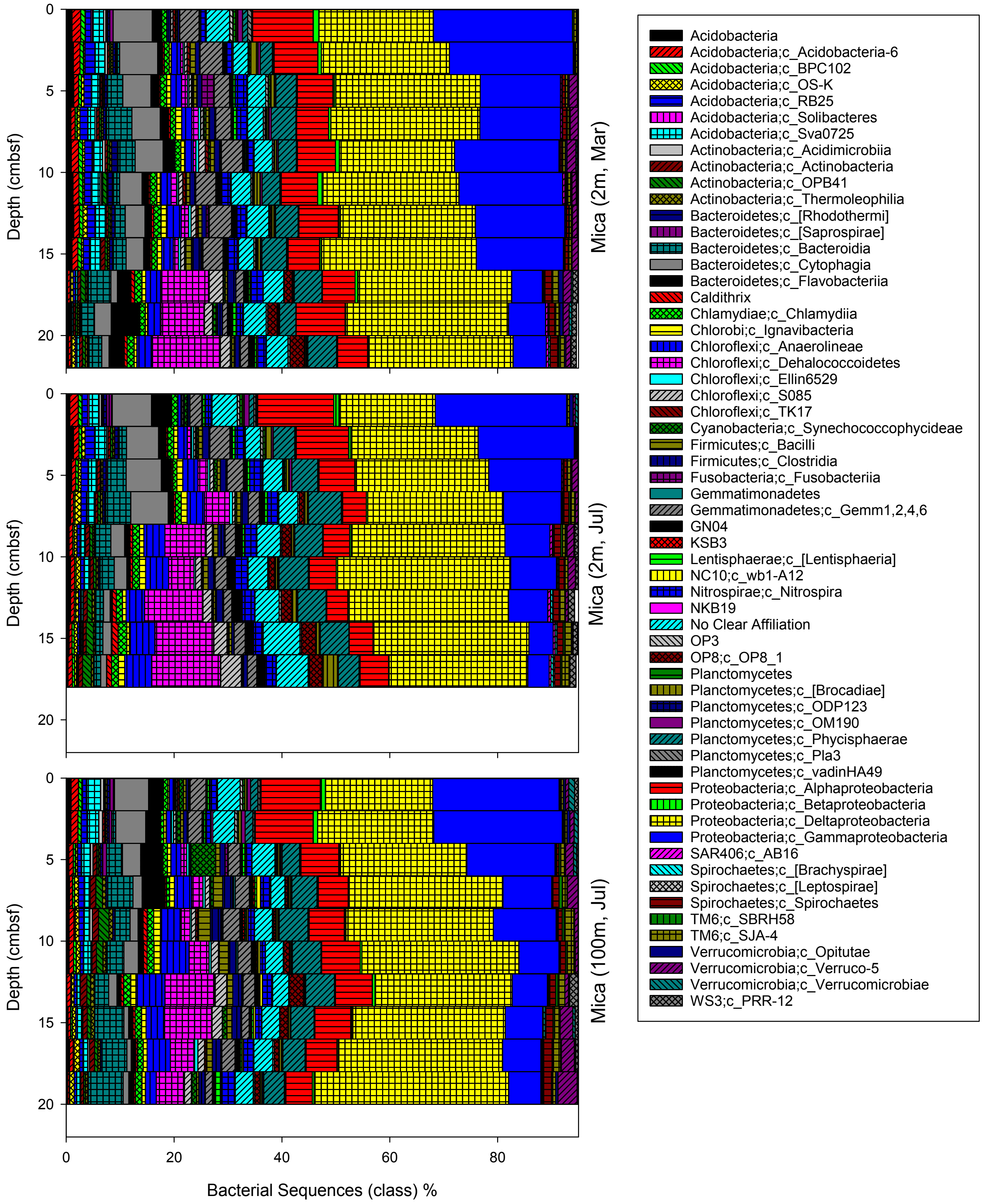
Depth (cmbsf)
Mica (2m, Mar)
Mica (2m, Jul)
Mica (100m, Jul)
0
5
10
15
20
0
20
40
60
80
Bacterial Sequences (class) %
Acidobacteria
Acidobacteria;c_Acidobacteria-6
Acidobacteria;c_BPC102
Acidobacteria;c_OS-K
Acidobacteria;c_RB25
Acidobacteria;c_Solibacteres
Acidobacteria;c_Sva0725
Actinobacteria;c_Acidimicrobiia
Actinobacteria;c_Actinobacteria
Actinobacteria;c_OPB41
Actinobacteria;c_Thermoleophilia
Bacteroidetes;c_[Rhodothermi]
Bacteroidetes;c_[Saprospirae]
Bacteroidetes;c_Bacteroidia
Bacteroidetes;c_Cytophagia
Bacteroidetes;c_Flavobacteriia
Caldithrix
Chlamydiae;c_Chlamydiia
Chlorobi;c_Ignavibacteria
Chloroflexi;c_Anaerolineae
Chloroflexi;c_Dehalococcoidetes
Chloroflexi;c_Ellin6529
Chloroflexi;c_S085
Chloroflexi;c_TK17
Cyanobacteria;c_Synechococcophycideae
Firmicutes;c_Bacilli
Firmicutes;c_Clostridia
Fusobacteria;c_Fusobacteriia
Gemmatimonadetes
Gemmatimonadetes;c_Gemm1,2,4,6
GN04
KSB3
Lentisphaerae;c_[Lentisphaeria]
NC10;c_wb1-A12
Nitrospirae;c_Nitrospira
NKB19
No Clear Affiliation
OP3
OP8;c_OP8_1
Planctomycetes
Planctomycetes;c_[Brocadiae]
Planctomycetes;c_ODP123
Planctomycetes;c_OM190
Planctomycetes;c_Phycisphaerae
Planctomycetes;c_Pla3
Planctomycetes;c_vadinHA49
Proteobacteria;c_Alphaproteobacteria
Proteobacteria;c_Betaproteobacteria
Proteobacteria;c_Deltaproteobacteria
Proteobacteria;c_Gammaproteobacteria
SAR406;c_AB16
Spirochaetes;c_[Brachyspirae]
Spirochaetes;c_[Leptospirae]
Spirochaetes;c_Spirochaetes
TM6;c_SBRH58
TM6;c_SJA-4
Verrucomicrobia;c_Opitutae
Verrucomicrobia;c_Verruco-5
Verrucomicrobia;c_Verrucomicrobiae
WS3;c_PRR-12

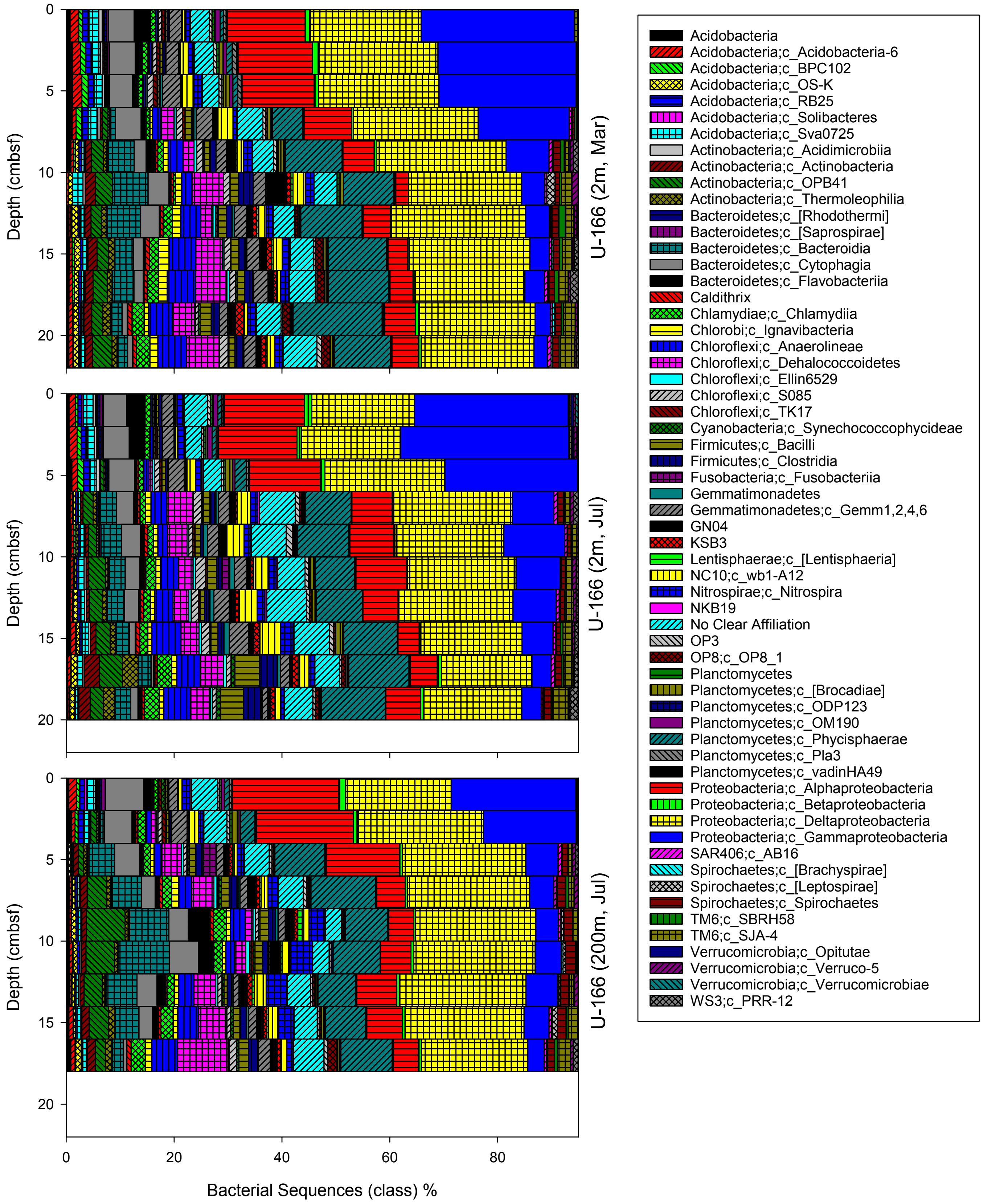
Depth (cmbsf)
0
5
10
15
20
U-166 (2m, Mar)
U-166 (2m, Jul)
U-166 (200m, Jul)
0
20
40
60
80
Bacterial Sequences (class) %
Acidobacteria
Acidobacteria;c_Acidobacteria-6
Acidobacteria;c_BPC102
Acidobacteria;c_OS-K
Acidobacteria;c_RB25
Acidobacteria;c_Solibacteres
Acidobacteria;c_Sva0725
Actinobacteria;c_Acidimicrobiia
Actinobacteria;c_Actinobacteria
Actinobacteria;c_OPB41
Actinobacteria;c_Thermoleophilia
Bacteroidetes;c_[Rhodothermi]
Bacteroidetes;c_[Saprospirae]
Bacteroidetes;c_Bacteroidia
Bacteroidetes;c_Cytophagia
Bacteroidetes;c_Flavobacteriia
Caldithrix
Chlamydiae;c_Chlamydiia
Chlorobi;c_Ignavibacteria
Chloroflexi;c_Anaerolineae
Chloroflexi;c_Dehalococcoidetes
Chloroflexi;c_Ellin6529
Chloroflexi;c_S085
Chloroflexi;c_TK17
Cyanobacteria;c_Synechococcophycideae
Firmicutes;c_Bacilli
Firmicutes;c_Clostridia
Fusobacteria;c_Fusobacteriia
Gemmatimonadetes
Gemmatimonadetes;c_Gemm1,2,4,6
GN04
KSB3
Lentisphaerae;c_[Lentisphaeria]
NC10;c_wb1-A12
Nitrospirae;c_Nitrospira
NKB19
No Clear Affiliation
OP3
OP8;c_OP8_1
Planctomycetes
Planctomycetes;c_[Brocadiae]
Planctomycetes;c_ODP123
Planctomycetes;c_OM190
Planctomycetes;c_Phycisphaerae
Planctomycetes;c_Pla3
Planctomycetes;c_vadinHA49
Proteobacteria;c_Alphaproteobacteria
Proteobacteria;c_Betaproteobacteria
Proteobacteria;c_Deltaproteobacteria
Proteobacteria;c_Gammaproteobacteria
SAR406;c_AB16
Spirochaetes;c_[Brachyspirae]
Spirochaetes;c_[Leptospirae]
Spirochaetes;c_Spirochaetes
TM6;c_SBRH58
TM6;c_SJA-4
Verrucomicrobia;c_Opitutae
Verrucomicrobia;c_Verruco-5
Verrucomicrobia;c_Verrucomicrobiae
WS3;c_PRR-12

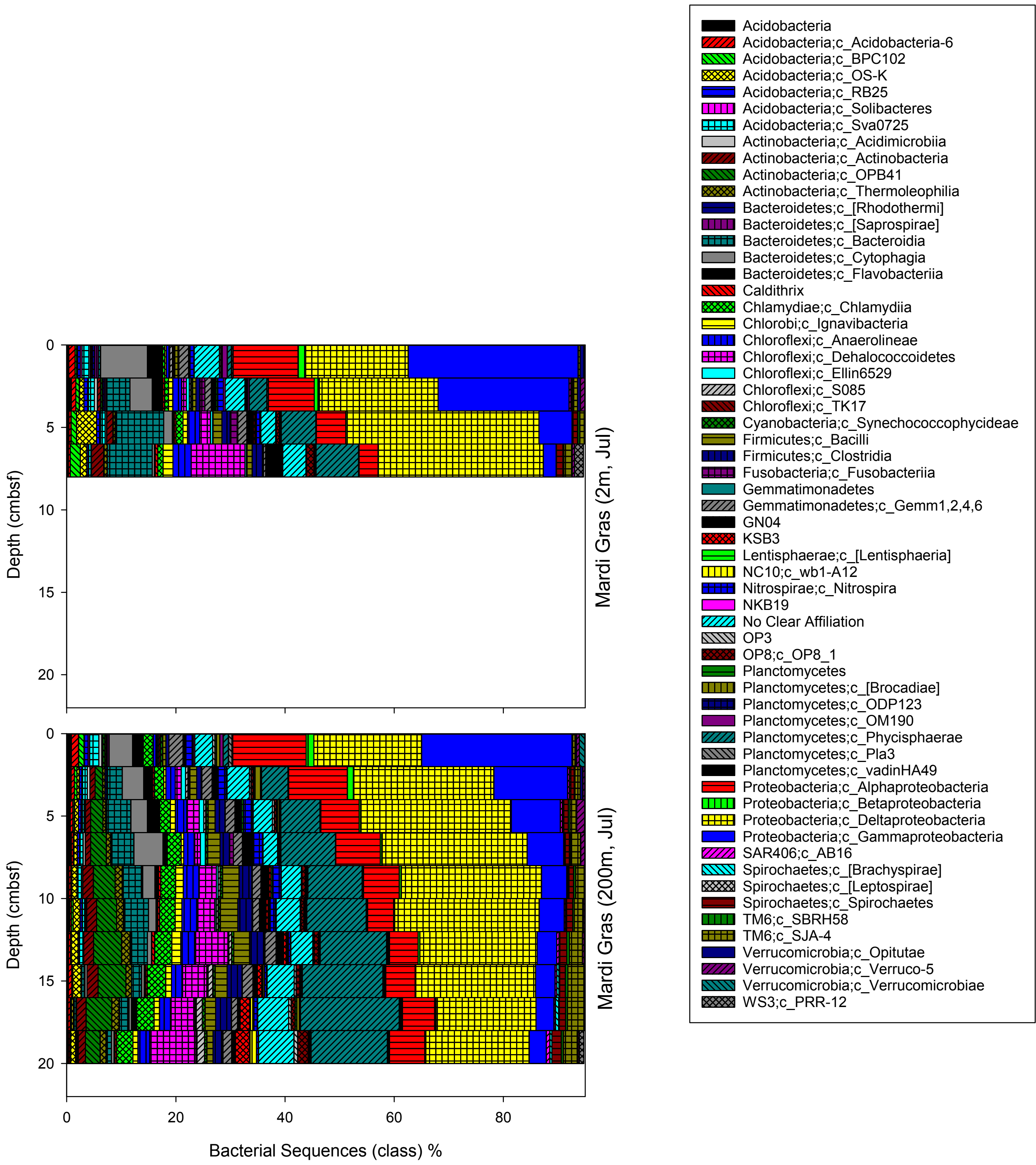
Depth (cmbsf)
Mardi Gras (2m, Jul)
Mardi Gras (200m, Jul)
Bacterial Sequences (class) %
0
5
10
15
20
0
20
40
60
80
Acidobacteria
Acidobacteria;c_Acidobacteria-6
Acidobacteria;c_BPC102
Acidobacteria;c_OS-K
Acidobacteria;c_RB25
Acidobacteria;c_Solibacteres
Acidobacteria;c_Sva0725
Actinobacteria;c_Acidimicrobiia
Actinobacteria;c_Actinobacteria
Actinobacteria;c_OPB41
Actinobacteria;c_Thermoleophilia
Bacteroidetes;c_[Rhodothermi]
Bacteroidetes;c_[Saprospirae]
Bacteroidetes;c_Bacteroidia
Bacteroidetes;c_Cytophagia
Bacteroidetes;c_Flavobacteriia
Caldithrix
Chlamydiae;c_Chlamydiia
Chlorobi;c_Ignavibacteria
Chloroflexi;c_Anaerolineae
Chloroflexi;c_Dehalococcoidetes
Chloroflexi;c_Ellin6529
Chloroflexi;c_S085
Chloroflexi;c_TK17
Cyanobacteria;c_Synechococcophycideae
Firmicutes;c_Bacilli
Firmicutes;c_Clostridia
Fusobacteria;c_Fusobacteriia
Gemmatimonadetes
Gemmatimonadetes;c_Gemm1,2,4,6
GN04
KSB3
Lentisphaerae;c_[Lentisphaeria]
NC10;c_wb1-A12
Nitrospirae;c_Nitrospira
NKB19
No Clear Affiliation
OP3
OP8;c_OP8_1
Planctomycetes
Planctomycetes;c_[Brocadiae]
Planctomycetes;c_ODP123
Planctomycetes;c_OM190
Planctomycetes;c_Phycisphaerae
Planctomycetes;c_Pla3
Planctomycetes;c_vadinHA49
Proteobacteria;c_Alphaproteobacteria
Proteobacteria;c_Betaproteobacteria
Proteobacteria;c_Deltaproteobacteria
Proteobacteria;c_Gammaproteobacteria
SAR406;c_AB16
Spirochaetes;c_[Brachyspirae]
Spirochaetes;c_[Leptospirae]
Spirochaetes;c_Spirochaetes
TM6;c_SBRH58
TM6;c_SJA-4
Verrucomicrobia;c_Opitutae
Verrucomicrobia;c_Verruco-5
Verrucomicrobia;c_Verrucomicrobiae
WS3;c_PRR-12

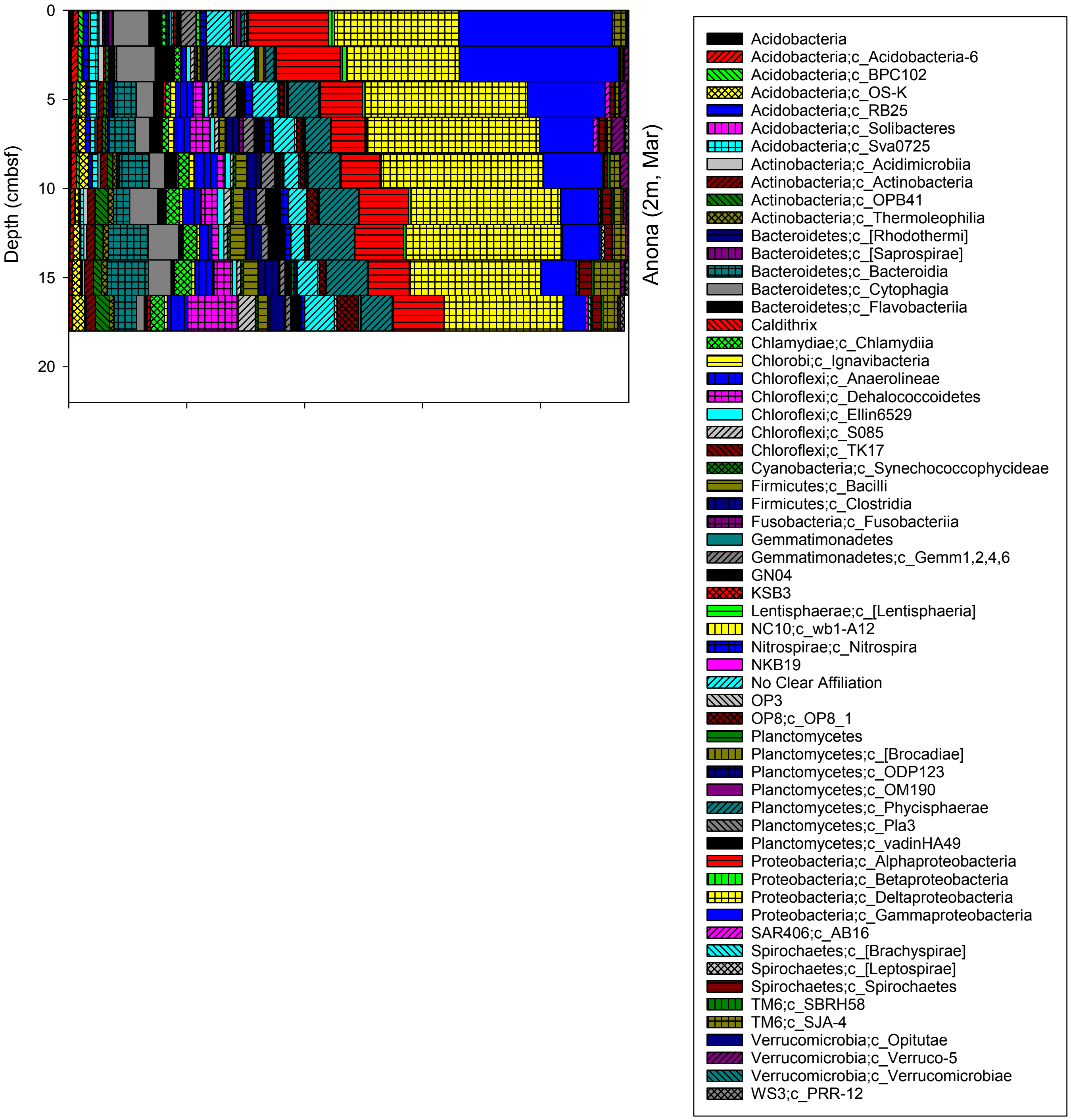

Depth (cmbsf)
0
5
10
15
20
Anona (2m, Mar)
Acidobacteria
Acidobacteria;c_Acidobacteria-6
Acidobacteria;c_BPC102
Acidobacteria;c_OS-K
Acidobacteria;c_RB25
Acidobacteria;c_Solibacteres
Acidobacteria;c_Sva0725
Actinobacteria;c_Acidimicrobiia
Actinobacteria;c_Actinobacteria
Actinobacteria;c_OPB41
Actinobacteria;c_Thermoleophilia
Bacteroidetes;c_[Rhodothermi]
Bacteroidetes;c_[Saprospirae]
Bacteroidetes;c_Bacteroidia
Bacteroidetes;c_Cytophagia
Bacteroidetes;c_Flavobacteriia
Caldithrix
Chlamydiae;c_Chlamydiia
Chlorobi;c_Ignavibacteria
Chloroflexi;c_Anaerolineae
Chloroflexi;c_Dehalococcoidetes
Chloroflexi;c_Ellin6529
Chloroflexi;c_S085
Chloroflexi;c_TK17
Cyanobacteria;c_Synechococcophycideae
Firmicutes;c_Bacilli
Firmicutes;c_Clostridia
Fusobacteria;c_Fusobacteriia
Gemmatimonadetes
Gemmatimonadetes;c_Gemm1,2,4,6
GN04
KSB3
Lentisphaerae;c_[Lentisphaeria]
NC10;c_wb1-A12
Nitrospirae;c_Nitrospira
NKB19
No Clear Affiliation
OP3
OP8;c_OP8_1
Planctomycetes
Planctomycetes;c_[Brocadiae]
Planctomycetes;c_ODP123
Planctomycetes;c_OM190
Planctomycetes;c_Phycisphaerae
Planctomycetes;c_Pla3
Planctomycetes;c_vadinHA49
Proteobacteria;c_Alphaproteobacteria
Proteobacteria;c_Betaproteobacteria
Proteobacteria;c_Deltaproteobacteria
Proteobacteria;c_Gammaproteobacteria
SAR406;c_AB16
Spirochaetes;c_[Brachyspirae]
Spirochaetes;c_[Leptospirae]
Spirochaetes;c_Spirochaetes
TM6;c_SBRH58
TM6;c_SJA-4
Verrucomicrobia;c_Opitutae
Verrucomicrobia;c_Verruco-5
Verrucomicrobia;c_Verrucomicrobiae
WS3;c_PRR-12

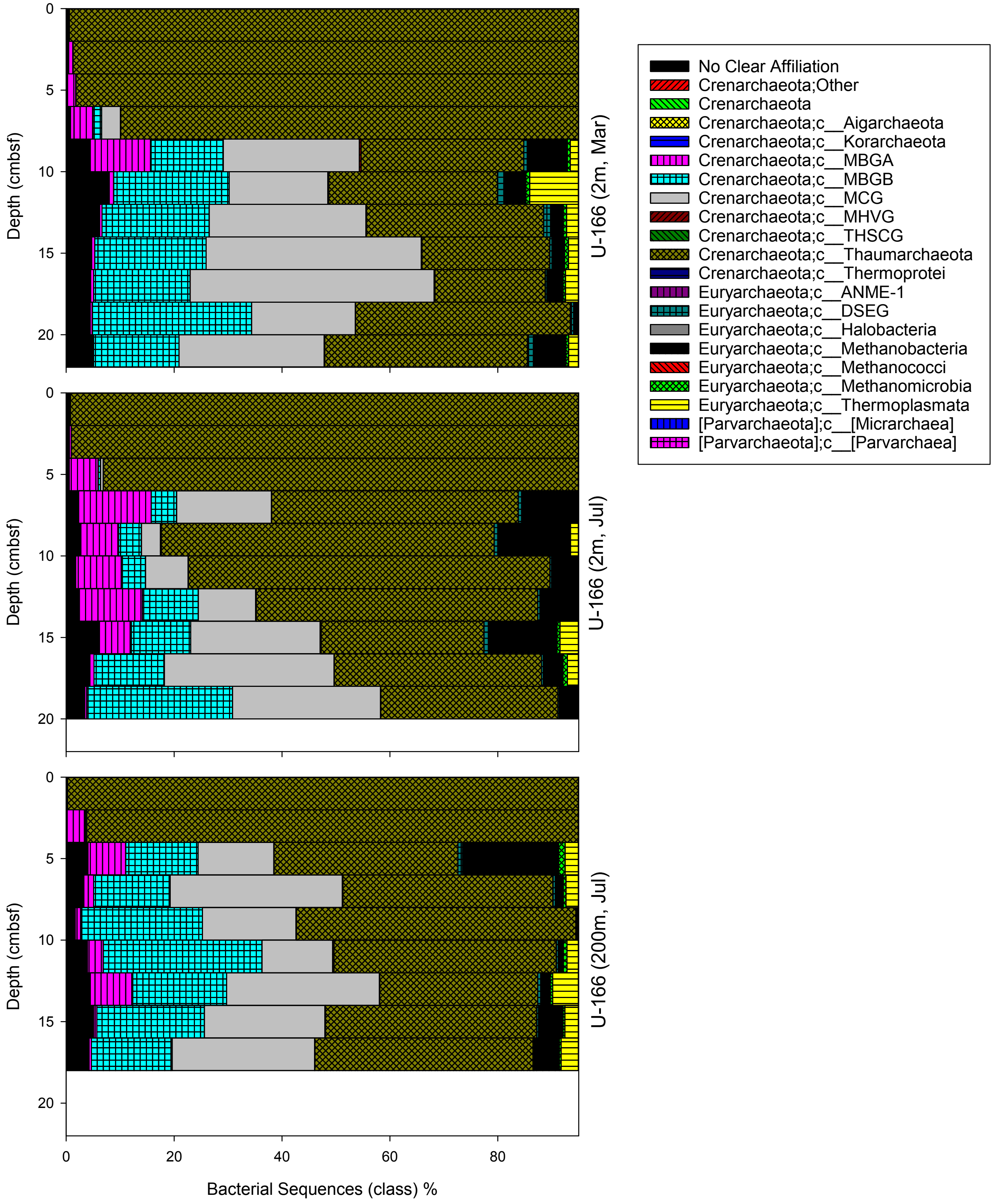
Depth (cmbsf)
U-166 (2m, Mar)
U-166 (2m, Jul)
U-166 (200m, Jul)
Bacterial Sequences (class) %
No Clear Affiliation
Crenarchaeota;Other
Crenarchaeota
Crenarchaeota;c__Aigarchaeota
Crenarchaeota;c__Korarchaeota
Crenarchaeota;c__MBGA
Crenarchaeota;c__MBGB
Crenarchaeota;c__MCG
Crenarchaeota;c__MHVG
Crenarchaeota;c__THSCG
Crenarchaeota;c__Thaumarchaeota
Crenarchaeota;c__Thermoprotei
Euryarchaeota;c__ANME-1
Euryarchaeota;c__DSEG
Euryarchaeota;c__Halobacteria
Euryarchaeota;c__Methanobacteria
Euryarchaeota;c__Methanococci
Euryarchaeota;c__Methanomicrobia
Euryarchaeota;c__Thermoplasmata
[Parvarchaeota];c__[Micrarchaea]
[Parvarchaeota];c__[Parvarchaea]

Figure S2.

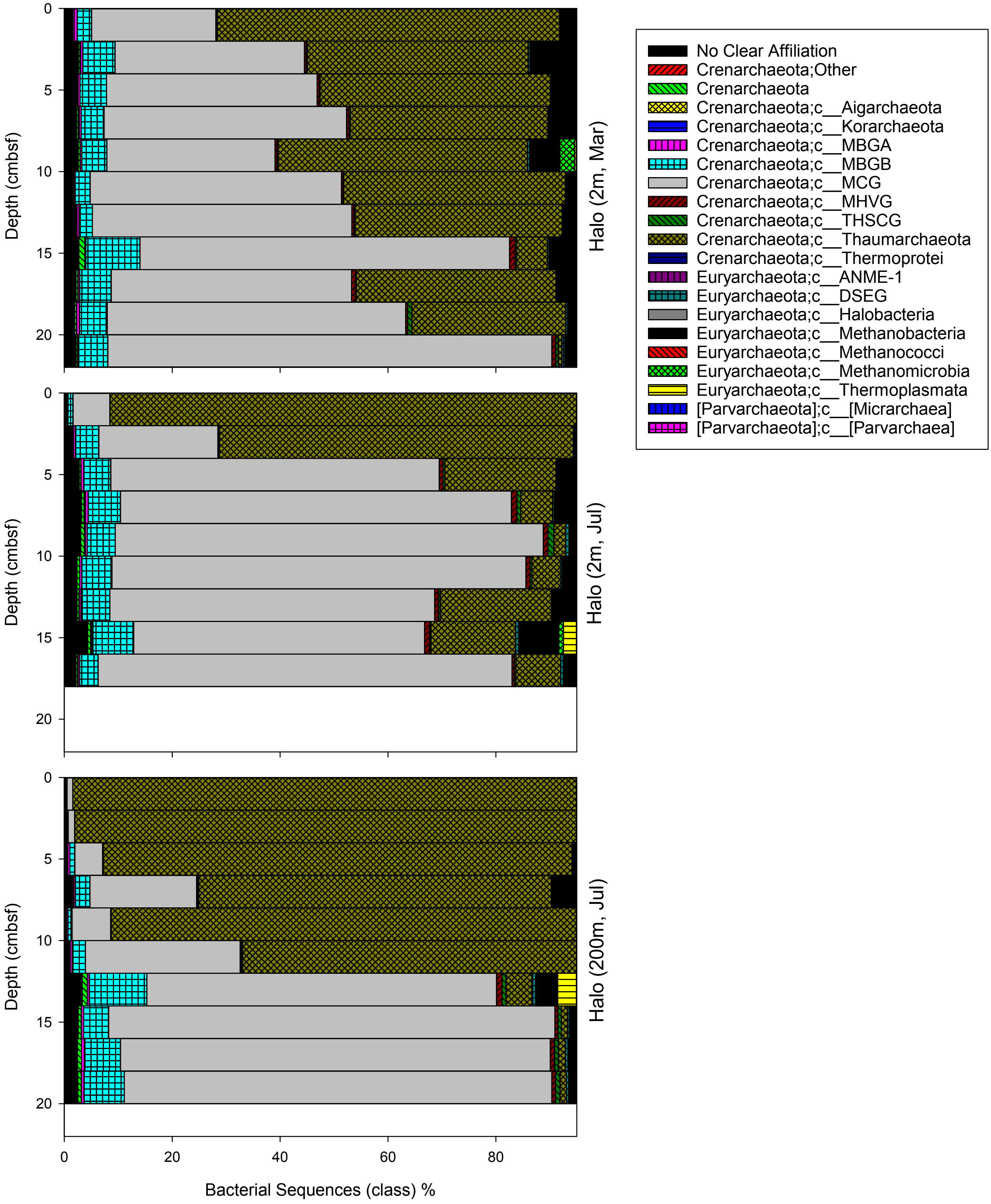

Depth (cmbsf)
Halo (2m, Mar)
Halo (2m, Jul)
Halo (200m, Jul)
Bacterial Sequences (class) %
0
20
40
60
80
5
10
15
No Clear Affiliation
Crenarchaeota;Other
Crenarchaeota
Crenarchaeota;c__Aigarchaeota
Crenarchaeota;c__Korarchaeota
Crenarchaeota;c__MBGA
Crenarchaeota;c__MBGB
Crenarchaeota;c__MCG
Crenarchaeota;c__MHVG
Crenarchaeota;c__THSCG
Crenarchaeota;c__Thaumarchaeota
Crenarchaeota;c__Thermoprotei
Euryarchaeota;c__ANME-1
Euryarchaeota;c__DSEG
Euryarchaeota;c__Halobacteria
Euryarchaeota;c__Methanobacteria
Euryarchaeota;c__Methanococci
Euryarchaeota;c__Methanomicrobia
Euryarchaeota;c__Thermoplasmata
[Parvarchaeota];c__[Micrarchaea]
[Parvarchaeota];c__[Parvarchaea]

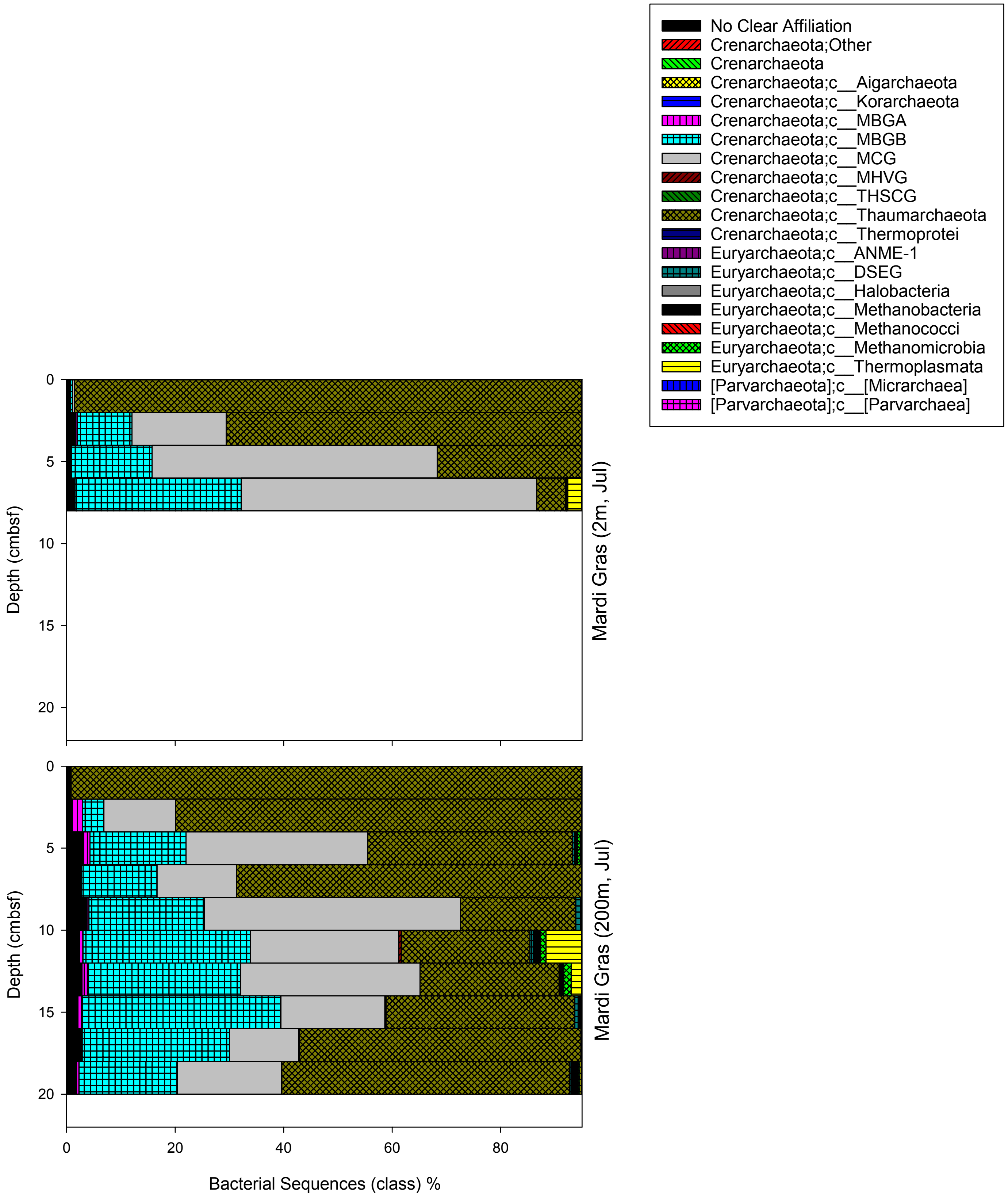

No Clear Affiliation
Crenarchaeota;Other
Crenarchaeota
Crenarchaeota;c__Aigarchaeota
Crenarchaeota;c__Korarchaeota
Crenarchaeota;c__MBGA
Crenarchaeota;c__MBGB
Crenarchaeota;c__MCG
Crenarchaeota;c__MHVG
Crenarchaeota;c__THSCG
Crenarchaeota;c__Thaumarchaeota
Crenarchaeota;c__Thermoprotei
Euryarchaeota;c__ANME-1
Euryarchaeota;c__DSEG
Euryarchaeota;c__Halobacteria
Euryarchaeota;c__Methanobacteria
Euryarchaeota;c__Methanococci
Euryarchaeota;c__Methanomicrobia
Euryarchaeota;c__Thermoplasmata
[Parvarchaeota];c__[Micrarchaea]
[Parvarchaeota];c__[Parvarchaea]
Depth (cmbsf)
Mardi Gras (2m, Jul)
Mardi Gras (200m, Jul)
0
5
10
15
20
0
20
40
60
80
Bacterial Sequences (class) %

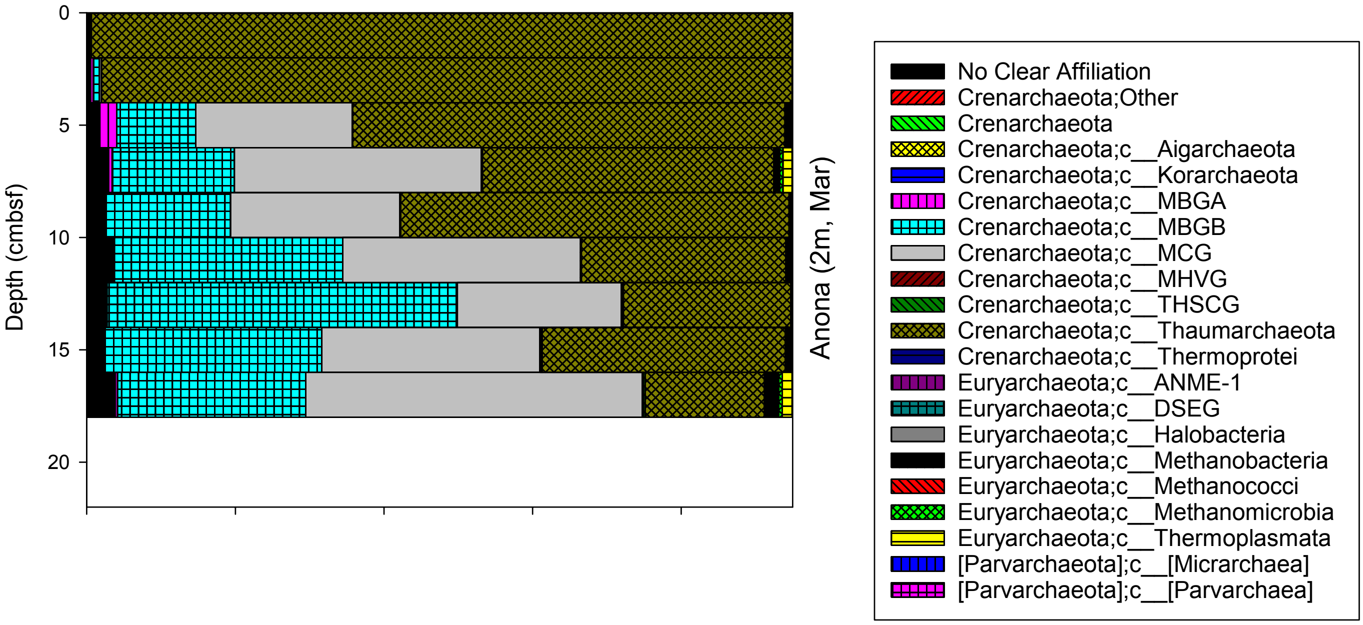

0
5
10
15
20
Depth (cmbsf)
Anona (2m, Mar)
No Clear Affiliation
Crenarchaeota;Other
Crenarchaeota
Crenarchaeota;c__Aigarchaeota
Crenarchaeota;c__Korarchaeota
Crenarchaeota;c__MBGA
Crenarchaeota;c__MBGB
Crenarchaeota;c__MCG
Crenarchaeota;c__MHVG
Crenarchaeota;c__THSCG
Crenarchaeota;c__Thaumarchaeota
Crenarchaeota;c__Thermoprotei
Euryarchaeota;c__ANME-1
Euryarchaeota;c__DSEG
Euryarchaeota;c__Halobacteria
Euryarchaeota;c__Methanobacteria
Euryarchaeota;c__Methanococci
Euryarchaeota;c__Methanomicrobia
Euryarchaeota;c__Thermoplasmata
[Parvarchaeota];c__[Micrarchaea]
[Parvarchaeota];c__[Parvarchaea]

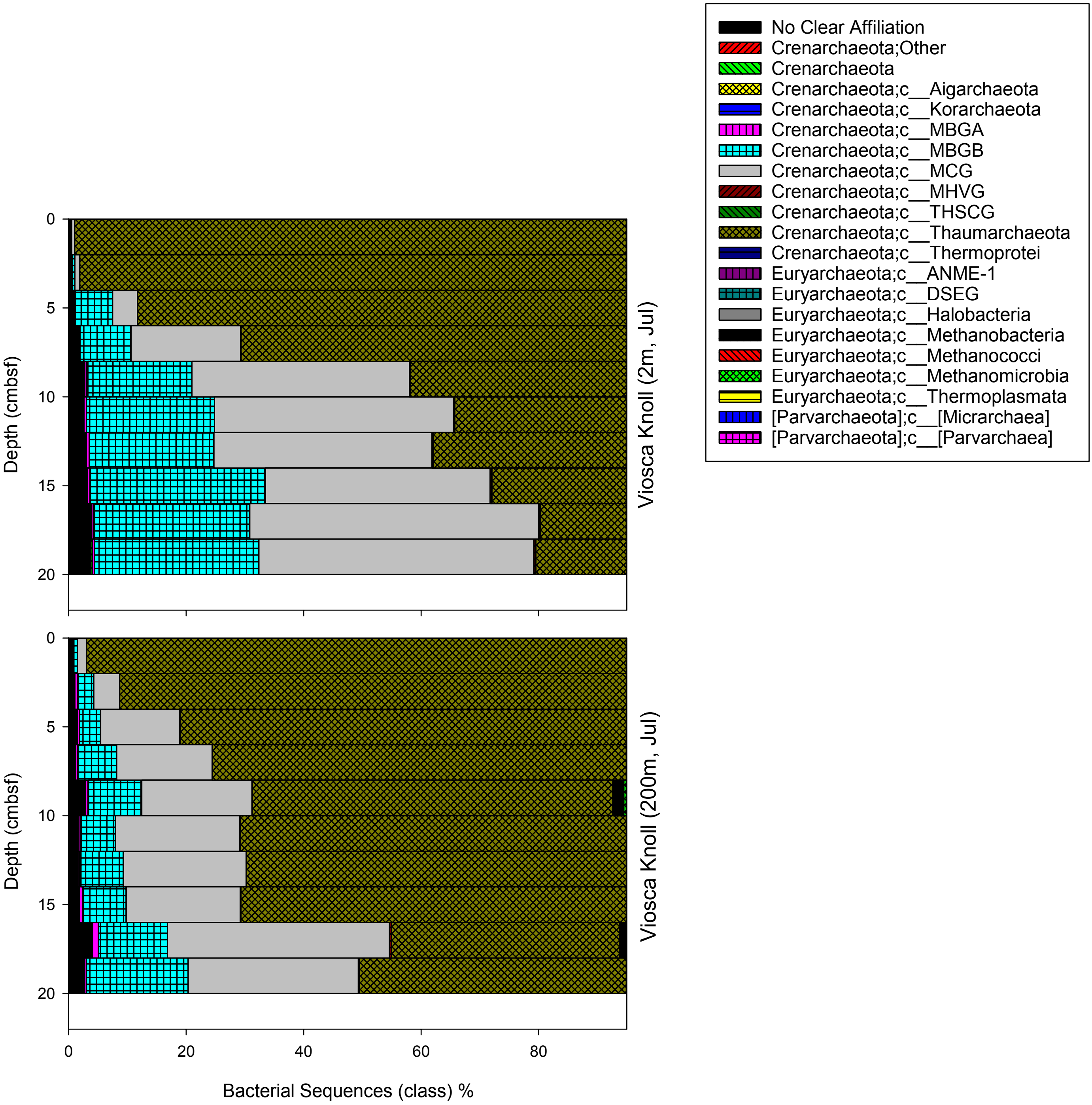

No Clear Affiliation
Crenarchaeota;Other
Crenarchaeota
Crenarchaeota;c__Aigarchaeota
Crenarchaeota;c__Korarchaeota
Crenarchaeota;c__MBGA
Crenarchaeota;c__MBGB
Crenarchaeota;c__MCG
Crenarchaeota;c__MHVG
Crenarchaeota;c__THSCG
Crenarchaeota;c__Thaumarchaeota
Crenarchaeota;c__Thermoprotei
Euryarchaeota;c__ANME-1
Euryarchaeota;c__DSEG
Euryarchaeota;c__Halobacteria
Euryarchaeota;c__Methanobacteria
Euryarchaeota;c__Methanococci
Euryarchaeota;c__Methanomicrobia
Euryarchaeota;c__Thermoplasmata
[Parvarchaeota];c__[Micrarchaea]
[Parvarchaeota];c__[Parvarchaea]
Depth (cmbsf)
0
5
10
15
20
Viosca Knoll (2m, Jul)
Viosca Knoll (200m, Jul)
0
20
40
60
80
Bacterial Sequences (class) %

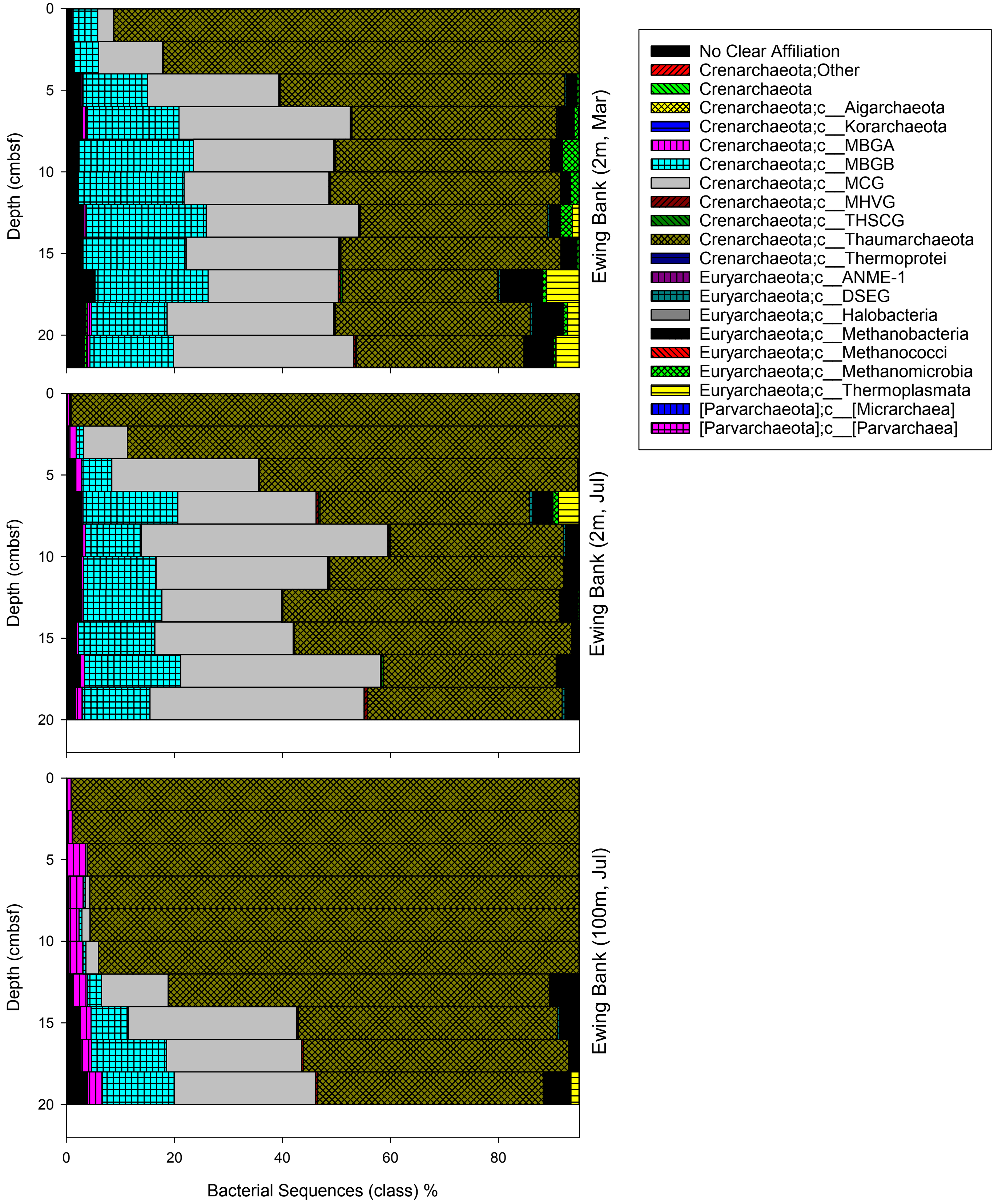

No Clear Affiliation
Crenarchaeota;Other
Crenarchaeota
Crenarchaeota;c__Aigarchaeota
Crenarchaeota;c__Korarchaeota
Crenarchaeota;c__MBGA
Crenarchaeota;c__MBGB
Crenarchaeota;c__MCG
Crenarchaeota;c__MHVG
Crenarchaeota;c__THSCG
Crenarchaeota;c__Thaumarchaeota
Crenarchaeota;c__Thermoprotei
Euryarchaeota;c__ANME-1
Euryarchaeota;c__DSEG
Euryarchaeota;c__Halobacteria
Euryarchaeota;c__Methanobacteria
Euryarchaeota;c__Methanococci
Euryarchaeota;c__Methanomicrobia
Euryarchaeota;c__Thermoplasmata
[Parvarchaeota];c__[Micrarchaea]
[Parvarchaeota];c__[Parvarchaea]
Depth (cmbsf)
Ewing Bank (2m, Mar)
Ewing Bank (2m, Jul)
Ewing Bank (100m, Jul)
0
5
10
15
20
0
20
40
60
80
Bacterial Sequences (class) %

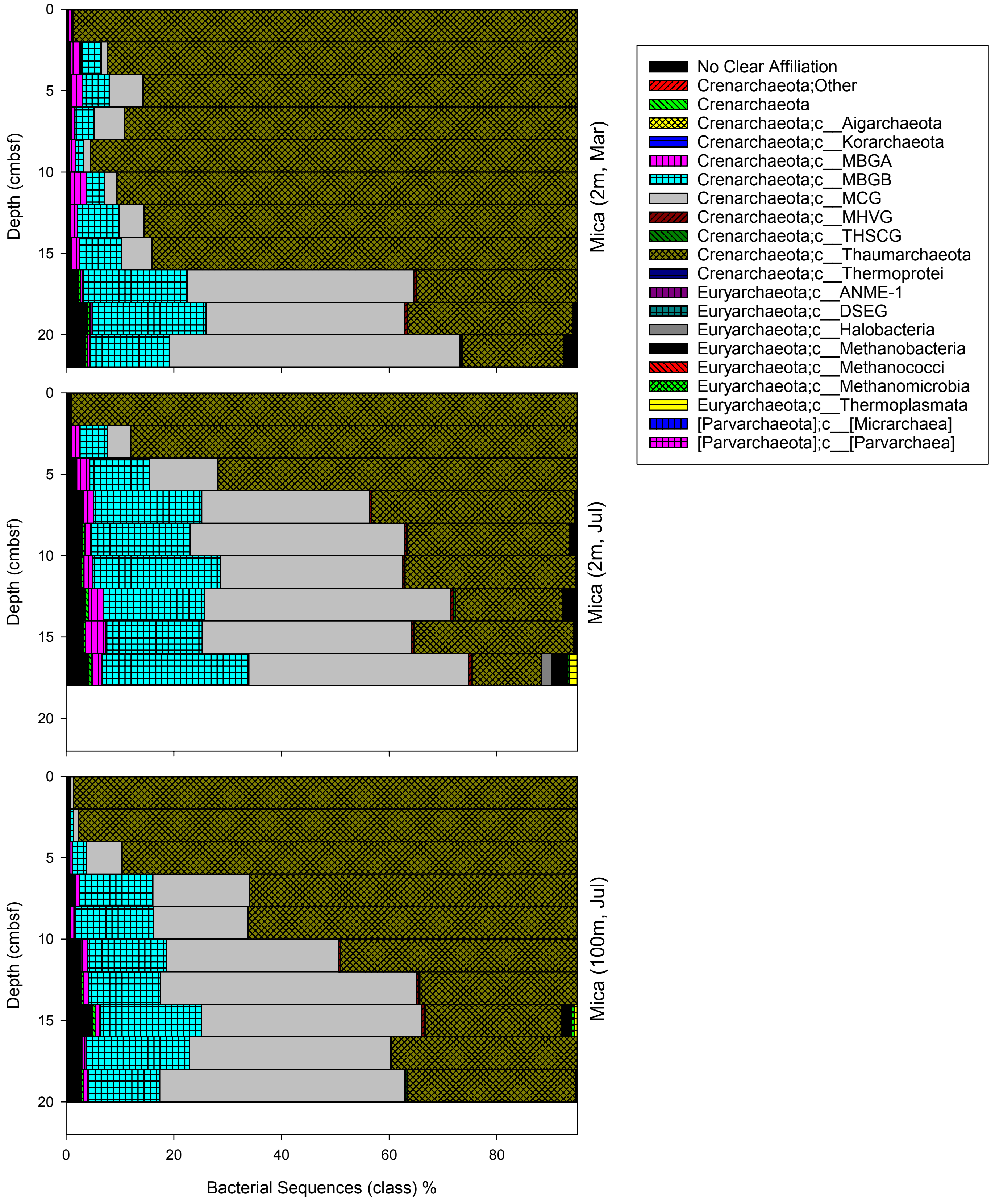

Depth (cmbsf)
Mica (2m, Mar)
Mica (2m, Jul)
Mica (100m, Jul)
0
5
10
15
20
0
20
40
60
80
Bacterial Sequences (class) %
No Clear Affiliation
Crenarchaeota;Other
Crenarchaeota
Crenarchaeota;c__Aigarchaeota
Crenarchaeota;c__Korarchaeota
Crenarchaeota;c__MBGA
Crenarchaeota;c__MBGB
Crenarchaeota;c__MCG
Crenarchaeota;c__MHVG
Crenarchaeota;c__THSCG
Crenarchaeota;c__Thaumarchaeota
Crenarchaeota;c__Thermoprotei
Euryarchaeota;c__ANME-1
Euryarchaeota;c__DSEG
Euryarchaeota;c__Halobacteria
Euryarchaeota;c__Methanobacteria
Euryarchaeota;c__Methanococci
Euryarchaeota;c__Methanomicrobia
Euryarchaeota;c__Thermoplasmata
[Parvarchaeota];c__[Micrarchaea]
[Parvarchaeota];c__[Parvarchaea]

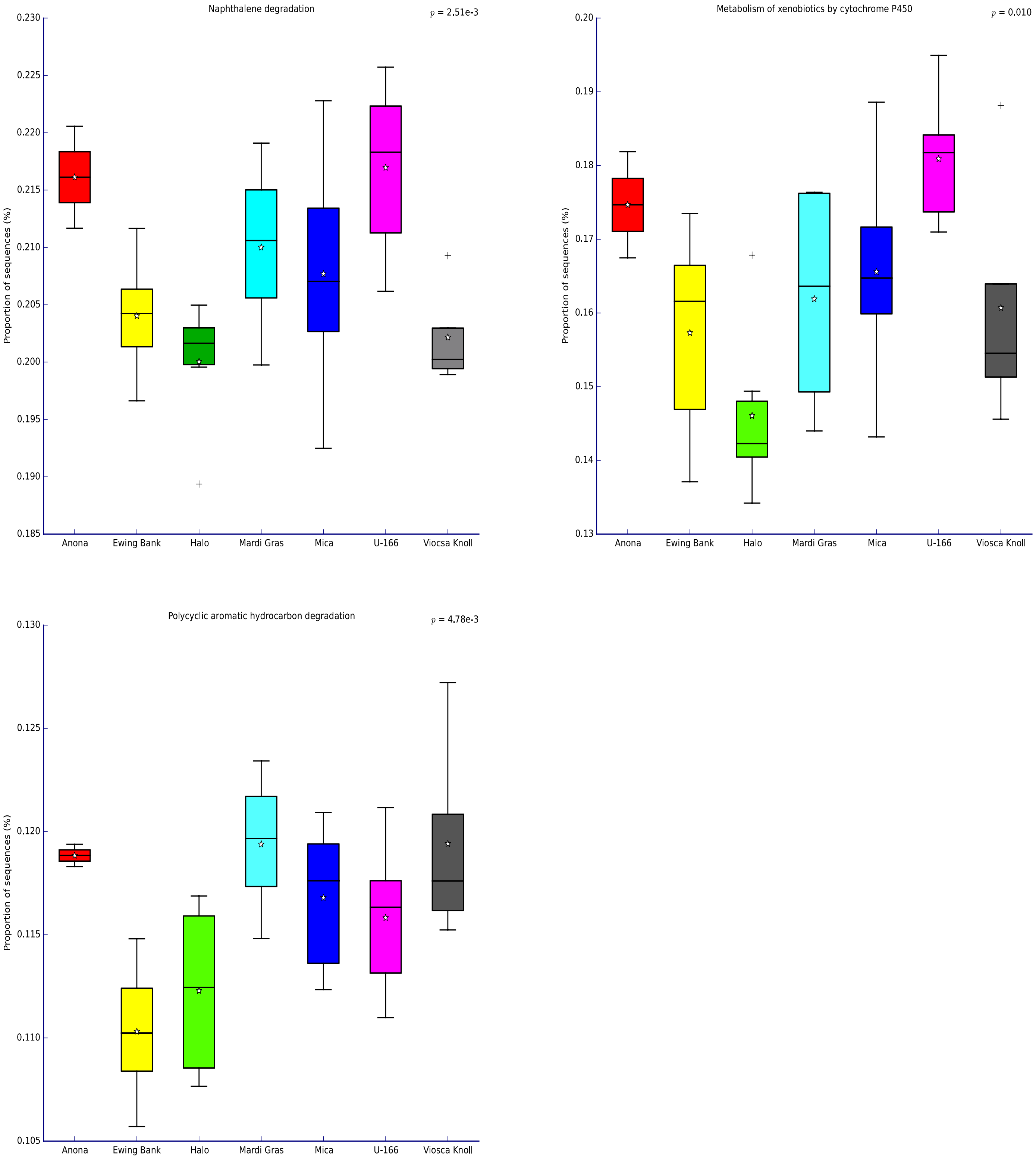
Naphthalene degradation
p = 2.51e-3
Metabolism of xenobiotics by cytochrome P450
p = 0.010
Polycyclic aromatic hydrocarbon degradation
p = 4.78e-3
Proportion of sequences (%)
Anona
Ewing Bank
Halo
Mardi Gras
Mica
U-166
Viocsa Knoll
Viosca Knoll

Figure S3.

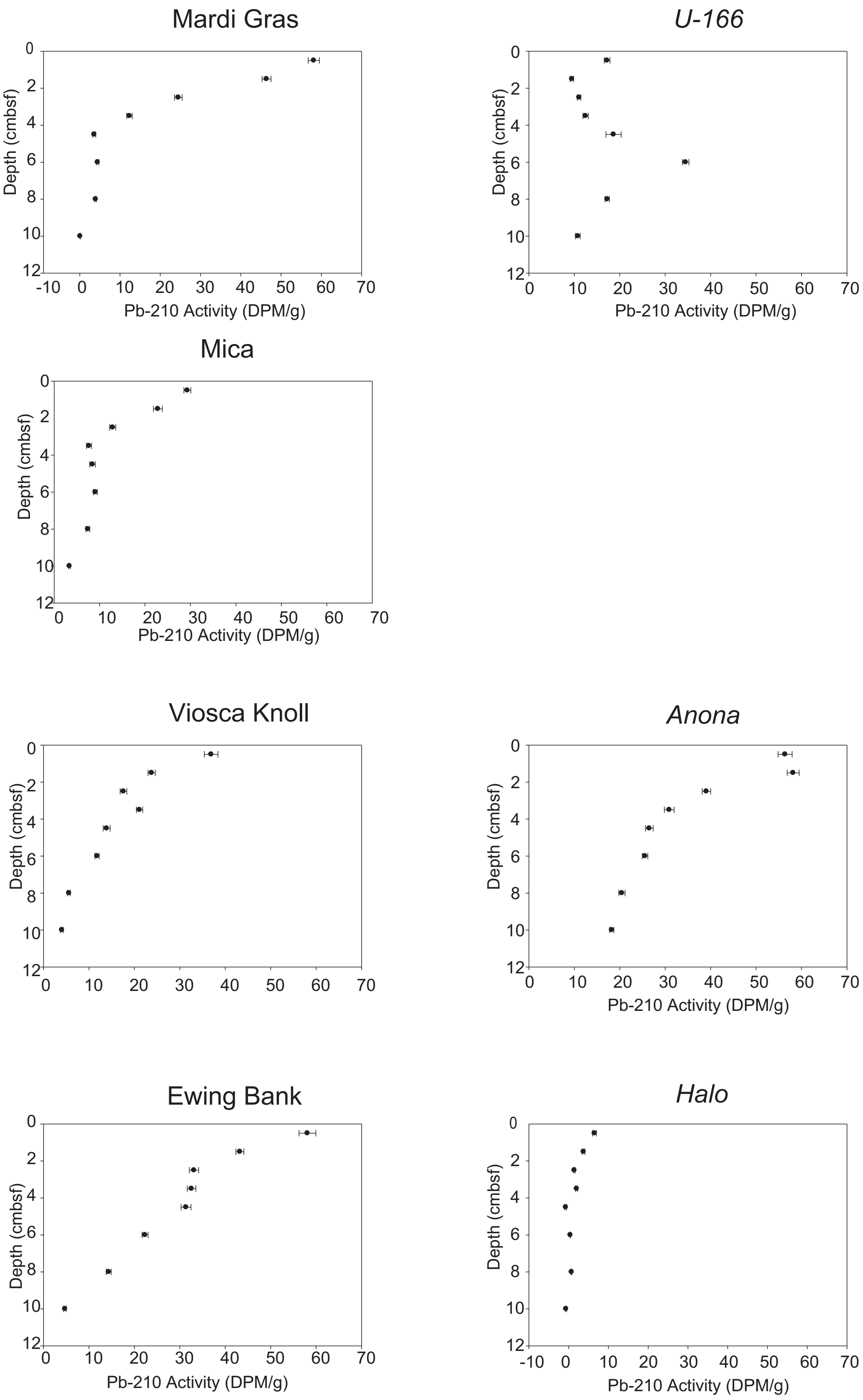
Mardi Gras
U-166
Mica
Viosca Knoll
Anona
Ewing Bank
Halo
Depth (cmbsf)
Pb-210 Activity (DPM/g)
0
2
4
6
8
10
12
-10
0
10
20
30
40
50
60
70

Figure S4.

Table S1. Coverage and richness indices for surface (0–10 cm below seafloor, cmbsf) and deep (11–22 cmbsf) sediment samples collected proximate to and away from shipwrecks.

		Near Shipwreck (1-2 m)				Away from Shipwreck (100-200 m)			
Shipwreck	Depth (cmbsf)	Good's Bacteria	Good's Archaea	Chao 1 Bacteria	Chao 1 Archaea	Good's Bacteria	Good's Archaea	Chao 1 Bacteria	Chao 1 Archaea
Halo	0-10	0.87	0.99	4447	1399	0.68	0.83	3071	766
	11-22	0.88	0.99	3749	1308	0.85	0.99	3096	1503
Ewing Bank	0-10	0.86	0.99	3512	731	0.67	0.83	1565	174
	11-22	0.90	0.99	2991	803	0.85	0.99	2415	924
Mica	0-10	0.86	1.00	3090	691	0.71	0.82	2655	449
	11-22	0.88	1.00	3130	859	0.85	0.99	2363	958
U-166	0-10	0.87	1.00	2460	466	0.70	0.83	1457	450
	11-22	0.85	0.99	2581	663	0.85	0.99	2243	779
Mardi Gras	0-10	0.66	0.79	1180	192	0.72	0.82	1899	248
	11-20	nd	nd	nd	nd	0.88	0.97	2484	381
Anona	0-10	0.88	1.00	3494	680	nd	nd	nd	nd
	11-18	0.90	0.91	1378	1359	nd	nd	nd	nd
Viosca Knoll	0-10	0.71	0.83	2320	457	0.72	0.83	2736	632
	11-20	0.87	0.99	3006	877	0.87	0.99	3928	896

Table S2. Similarity Percent (SIMPER) analysis, identifying bacterial drivers of within group similarity, and between group dissimilarity.

Group *Halo*
Average similarity: 71.68

Species	Av.Abund	Av.Sim	Sim/SD	Contrib%	Cum.%
p_Proteobacteria;c_Gammaproteobacteria;o_Thiotrichales;f_Piscirickettsiaceae;g_;s_	0.25	1.51	3.33	2.11	2.11
Unassigned;Other;Other;Other;Other;Other;Other	0.2	1.35	8.13	1.88	3.99
p_Proteobacteria;c_Deltaproteobacteria;o_Myxococcales;f_;g_;s_	0.16	1.07	15.49	1.49	5.48
p_Proteobacteria;c_Deltaproteobacteria;o_NB1-j;f_NB1-i;g_;s_	0.16	1.03	5.9	1.44	6.92
p_Proteobacteria;c_Deltaproteobacteria;o_NB1-j;f_;g_;s_	0.15	1.03	12.96	1.44	8.36
p_Proteobacteria;c_Alphaproteobacteria;o_;f_;g_;s_	0.16	1.01	4.42	1.41	9.77
p_Acidobacteria;c_Sva0725;o_Sva0725;f_;g_;s_	0.14	0.92	6.03	1.28	11.05

Group Mica
Average similarity: 69.65

Species	Av.Abund	Av.Sim	Sim/SD	Contrib%	Cum.%
p_Proteobacteria;c_Gammaproteobacteria;o_Thiotrichales;f_Piscirickettsiaceae;g_;s_	0.29	1.9	4.25	2.73	2.73
p_Proteobacteria;c_Deltaproteobacteria;o_NB1-j;f_NB1-i;g_;s_	0.22	1.55	9.94	2.22	4.95
p_Bacteroidetes;c_Cytophagia;o_Cytophagales;f_Flammeovirgaceae;g_;s_	0.21	1.4	3.84	2.01	6.96
Unassigned;Other;Other;Other;Other;Other;Other	0.18	1.3	13.2	1.86	8.82
p_Proteobacteria;c_Deltaproteobacteria;o_Myxococcales;f_;g_;s_	0.14	1.03	16.54	1.48	10.3

Group *U-166*
Average similarity: 64.24

Species	Av.Abund	Av.Sim	Sim/SD	Contrib%	Cum.%
p_Proteobacteria;c_Gammaproteobacteria;o_Thiotrichales;f_Piscirickettsiaceae;g_;s_	0.29	1.91	2.46	2.97	2.97
p_Proteobacteria;c_Deltaproteobacteria;o_NB1-j;f_NB1-i;g_;s_	0.24	1.88	9.62	2.92	5.89
Unassigned;Other;Other;Other;Other;Other;Other	0.2	1.52	9.58	2.36	8.26
p_Bacteroidetes;c_Cytophagia;o_Cytophagales;f_Flammeovirgaceae;g_;s_	0.2	1.51	6.86	2.36	10.61

Group *Anona*
Average similarity: 68.20

Species	Av.Abund	Av.Sim	Sim/SD	Contrib%	Cum.%
p_Proteobacteria;c_Gammaproteobacteria;o_Thiotrichales;f_Piscirickettsiaceae;g_;s_	0.27	1.59	3.45	2.33	2.33
p_Proteobacteria;c_Deltaproteobacteria;o_NB1-j;f_NB1-i;g_;s_	0.2	1.42	16.06	2.08	4.41
Unassigned;Other;Other;Other;Other;Other;Other	0.19	1.3	7.82	1.91	6.32
p_Bacteroidetes;c_Cytophagia;o_Cytophagales;f_Flammeovirgaceae;g_;s_	0.19	1.21	5.74	1.77	8.09
p_Proteobacteria;c_Deltaproteobacteria;o_NB1-j;f_;g_;s_	0.16	1.1	13.77	1.61	9.7
p_Proteobacteria;c_Alphaproteobacteria;o_;f_;g_;s_	0.17	1.04	3.81	1.53	11.23

Group Ewing Bank
Average similarity: 64.08

Species	Av.Abund	Av.Sim	Sim/SD	Contrib%	Cum.%
p_Proteobacteria;c_Gammaproteobacteria;o_Thiotrichales;f_Piscirickettsiaceae;g_;s_	0.27	1.59	1.6	2.47	2.47
Unassigned;Other;Other;Other;Other;Other;Other	0.2	1.49	11.4	2.32	4.79
p_Proteobacteria;c_Deltaproteobacteria;o_NB1-j;f_NB1-i;g_;s_	0.21	1.47	4.79	2.29	7.09
p_Proteobacteria;c_Alphaproteobacteria;o_;f_;g_;s_	0.18	1.21	3.2	1.89	8.98
p_Proteobacteria;c_Deltaproteobacteria;o_NB1-j;f_;g_;s_	0.16	1.21	7.67	1.88	10.86

Group Viosca Knoll
Average similarity: 74.17

Species	Av.Abund	Av.Sim	Sim/SD	Contrib%	Cum.%
p_Proteobacteria;c_Gammaproteobacteria;o_Thiotrichales;f_Piscirickettsiaceae;g_;s_	0.33	2.24	6.18	3.02	3.02
p_Proteobacteria;c_Deltaproteobacteria;o_NB1-j;f_NB1-i;g_;s_	0.2	1.47	13.08	1.98	5
p_Proteobacteria;c_Deltaproteobacteria;o_NB1-j;f_;g_;s_	0.17	1.23	19.71	1.65	6.66
p_Proteobacteria;c_Deltaproteobacteria;o_Myxococcales;f_;g_;s_	0.17	1.22	13.46	1.65	8.3
Unassigned;Other;Other;Other;Other;Other;Other	0.17	1.21	9.3	1.63	9.93
p_Proteobacteria;c_Alphaproteobacteria;o_;f_;g_;s_	0.17	1.21	8.55	1.63	11.56

Group Mardi Gras
Average similarity: 61.44

Species	Av.Abund	Av.Sim	Sim/SD	Contrib%	Cum.%
p_Proteobacteria;c_Deltaproteobacteria;o_NB1-j;f_NB1-i;g_;s_	0.22	1.59	5.86	2.59	2.59
Unassigned;Other;Other;Other;Other;Other;Other	0.18	1.42	10.69	2.31	4.9
p_Proteobacteria;c_Gammaproteobacteria;o_Thiotrichales;f_Piscirickettsiaceae;g_;s_	0.23	1.21	1.68	1.96	6.86
p_Planctomycetes;c_Phycisphaerae;o_MSBL9;f_;g_;s_	0.2	1.2	1.62	1.95	8.81
p_Bacteroidetes;c_Cytophagia;o_Cytophagales;f_Flammeovirgaceae;g_;s_	0.17	1.13	2.72	1.84	10.65

Groups *Halo* & Mica
Average dissimilarity = 33.67

Species	Group *Halo* Av.Abund	Group Mica Av.Abund	Av.Diss	Diss/SD	Contrib%	Cum.%
p_Chloroflexi;c_Dehalococcoidetes;o_Dehalococcoidales;f_Dehalococcoidaceae;g_;s_	0.14	0.09	0.37	1	1.09	1.09
p_Proteobacteria;c_Gammaproteobacteria;o_Thiotrichales;f_Piscirickettsiaceae;g_;s_	0.25	0.29	0.31	1.27	0.91	2
p_Bacteroidetes;c_Cytophagia;o_Cytophagales;f_Flammeovirgaceae;g_;s_	0.15	0.21	0.28	1.67	0.83	2.83
p_Planctomycetes;c_Phycisphaerae;o_MSBL9;f_;g_;s_	0.11	0.16	0.25	1.62	0.73	3.56
p_Bacteroidetes;c_Bacteroidia;o_Bacteroidales;f_;g_;s_	0.11	0.12	0.23	1.63	0.68	4.24
p_Proteobacteria;c_Deltaproteobacteria;o_NB1-j;f_NB1-i;g_;s_	0.16	0.22	0.22	1.62	0.64	4.88
p_Proteobacteria;c_Deltaproteobacteria;o_Desulfobacterales;f_Desulfobulbaceae;g_;s_	0.12	0.1	0.2	1.37	0.61	5.48
p_Proteobacteria;c_Deltaproteobacteria;o_Desulfobacterales;f_Desulfobacteraceae;g_Desulfococcus;s_	0.14	0.13	0.2	1.52	0.59	6.07
p_Proteobacteria;c_Deltaproteobacteria;o_;f_;g_;s_	0.15	0.12	0.2	1.33	0.59	6.66
p_OP8;c_OP8_1;o_;f_;g_;s_	0.07	0.03	0.2	1.05	0.58	7.24

p_Fusobacteria;c_Fusobacteriia;o_Fusobacteriales;f_Fusobacteriaceae;g_Propionigenium;s_	0.07	0.02	0.2	2.25	0.58	7.82
p_Proteobacteria;c_Deltaproteobacteria;o_Desulfobacterales;f_Desulfobacteraceae;g_;s_	0.1	0.07	0.19	1.39	0.57	8.4
p_Lentisphaerae;c_[Lentisphaeria];o_Lentisphaerales;f_;g_;s_	0.08	0.03	0.19	1.75	0.55	8.95
p_Proteobacteria;c_Gammaproteobacteria;o_Oceanospirillales;f_Endozoicimonaceae;g_;s_	0.06	0	0.19	1.34	0.55	9.5

Groups *Halo* & *U-166*
Average dissimilarity = 38.90

Species	Group *Halo* Av.Abund	Group *U-166* Av.Abund	Av.Diss	Diss/SD	Contrib%	Cum.%
p_Chloroflexi;c_Dehalococcoidetes;o_Dehalococcoidales;f_Dehalococcoidaceae;g_;s_	0.14	0.08	0.41	1.04	1.05	1.05
p_Proteobacteria;c_Gammaproteobacteria;o_Thiotrichales;f_Piscirickettsiaceae;g_;s_	0.25	0.29	0.41	1.53	1.05	2.1
p_Planctomycetes;c_Phycisphaerae;o_MSBL9;f_;g_;s_	0.11	0.16	0.4	1.49	1.03	3.12
p_NC10;c_wb1-A12;o_;f_;g_;s_	0.02	0.11	0.35	2.37	0.91	4.03
p_Bacteroidetes;c_Bacteroidia;o_Bacteroidales;f_;g_;s_	0.11	0.11	0.32	1.94	0.83	4.86
p_Proteobacteria;c_Alphaproteobacteria;o_Rhodobacterales;f_Rhodobacteraceae;g_;s_	0.12	0.2	0.31	1.37	0.8	5.66
p_Proteobacteria;c_Deltaproteobacteria;o_NB1-j;f_NB1-i;g_;s_	0.16	0.24	0.29	2.24	0.76	6.42
p_Proteobacteria;c_Deltaproteobacteria;o_Desulfobacterales;f_Desulfobacteraceae;g_Desulfococcus;s_	0.14	0.11	0.28	1.59	0.73	7.15
p_Proteobacteria;c_Deltaproteobacteria;o_;f_;g_;s_	0.15	0.09	0.27	1.49	0.7	7.85
p_Proteobacteria;c_Deltaproteobacteria;o_Desulfobacterales;f_Desulfobulbaceae;g_;s_	0.12	0.06	0.26	1.64	0.67	8.52
p_Lentisphaerae;c_[Lentisphaeria];o_Lentisphaerales;f_;g_;s_	0.08	0.01	0.25	2.25	0.65	9.17

Groups Mica & *U-166*
Average dissimilarity = 34.96

Species	Group Mica Av.Abund	Group *U-166* Av.Abund	Av.Diss	Diss/SD	Contrib%	Cum.%
p_Planctomycetes;c_Phycisphaerae;o_MSBL9;f_;g_;s_	0.16	0.16	0.42	1.77	1.2	1.2
p_Proteobacteria;c_Gammaproteobacteria;o_Thiotrichales;f_Piscirickettsiaceae;g_;s_	0.29	0.29	0.38	1.45	1.1	2.3
p_Bacteroidetes;c_Bacteroidia;o_Bacteroidales;f_;g_;s_	0.12	0.11	0.37	1.51	1.05	3.35
p_Proteobacteria;c_Deltaproteobacteria;o_Desulfobacterales;f_Desulfobacteraceae;g_Desulfococcus;s_	0.13	0.11	0.32	1.51	0.9	4.25
p_NC10;c_wb1-A12;o_;f_;g_;s_	0.04	0.11	0.3	1.93	0.87	5.12
p_Chloroflexi;c_Dehalococcoidetes;o_Dehalococcoidales;f_Dehalococcoidaceae;g_;s_	0.09	0.08	0.3	1.4	0.84	5.96
p_Proteobacteria;c_Alphaproteobacteria;o_;f_;g_;s_	0.14	0.19	0.28	1.39	0.81	6.78
p_Proteobacteria;c_Alphaproteobacteria;o_Rhodobacterales;f_Rhodobacteraceae;g_;s_	0.16	0.2	0.25	1.34	0.72	7.5
p_Actinobacteria;c_OPB41;o_;f_;g_;s_	0.06	0.1	0.24	1.13	0.69	8.19
p_Proteobacteria;c_Deltaproteobacteria;o_Desulfobacterales;f_Desulfobulbaceae;g_;s_	0.1	0.06	0.23	1.43	0.66	8.86
p_Nitrospirae;c_Nitrospira;o_Nitrospirales;f_[Thermodesulfovibrionaceae];g_;s_	0.07	0.04	0.21	1.52	0.59	9.45
p_Proteobacteria;c_Gammaproteobacteria;o_Chromatiales;f_Ectothiorhodospiraceae;g_;s_	0.09	0.11	0.2	1.5	0.57	10.02

Groups *Halo* & *Anona*
Average dissimilarity = 34.63

Species	Group *Halo* Av.Abund	Group *Anona* Av.Abund	Av.Diss	Diss/SD	Contrib%	Cum.%
p_Chloroflexi;c_Dehalococcoidetes;o_Dehalococcoidales;f_Dehalococcoidaceae;g_;s_	0.14	0.08	0.38	1	1.09	1.09
p_Proteobacteria;c_Gammaproteobacteria;o_Thiotrichales;f_Piscirickettsiaceae;g_;s_	0.25	0.27	0.33	1.43	0.95	2.04
p_Bacteroidetes;c_Bacteroidia;o_Bacteroidales;f_;g_;s_	0.11	0.14	0.29	2.2	0.82	2.86
p_Planctomycetes;c_Phycisphaerae;o_MSBL9;f_;g_;s_	0.11	0.16	0.28	1.75	0.81	3.68
p_Proteobacteria;c_Deltaproteobacteria;o_Desulfobacterales;f_Desulfobacteraceae;g_Desulfococcus;s_	0.14	0.15	0.28	2.2	0.8	4.48
p_Proteobacteria;c_Deltaproteobacteria;o_Desulfobacterales;f_Desulfobulbaceae;g_;s_	0.12	0.13	0.25	1.49	0.72	5.2
p_TM6;c_SJA-4;o_;f_;g_;s_	0.04	0.1	0.22	1.76	0.64	5.84
p_Fusobacteria;c_Fusobacteriia;o_Fusobacteriales;f_Fusobacteriaceae;g_Propionigenium;s_	0.07	0.01	0.22	2.81	0.64	6.47
p_Proteobacteria;c_Deltaproteobacteria;o_;f_;g_;s_	0.15	0.11	0.22	1.37	0.63	7.1
p_Lentisphaerae;c_[Lentisphaeria];o_Lentisphaerales;f_;g_;s_	0.08	0.02	0.21	2.1	0.62	7.71
p_Bacteroidetes;c_Cytophagia;o_Cytophagales;f_Flammeovirgaceae;g_;s_	0.15	0.19	0.21	1.39	0.61	8.32
p_Proteobacteria;c_Deltaproteobacteria;o_Desulfobacterales;f_Desulfobacteraceae;g_;s_	0.1	0.09	0.19	1.31	0.55	8.87
p_Proteobacteria;c_Alphaproteobacteria;o_;f_;g_;s_	0.16	0.17	0.19	1.49	0.54	9.41

Groups *Halo* & Ewing Bank
Average dissimilarity = 37.09

Species	Group *Halo* Av.Abund	Group Ewing Bank Av.Abund	Av.Diss	Diss/SD	Contrib%	Cum.%
p_Chloroflexi;c_Dehalococcoidetes;o_Dehalococcoidales;f_Dehalococcoidaceae;g_;s_	0.14	0.15	0.47	1.24	1.27	1.27
p_Proteobacteria;c_Gammaproteobacteria;o_Thiotrichales;f_Piscirickettsiaceae;g_;s_	0.25	0.27	0.46	1.41	1.24	2.51
p_Planctomycetes;c_Phycisphaerae;o_MSBL9;f_;g_;s_	0.11	0.16	0.32	1.71	0.87	3.38
p_NC10;c_wb1-A12;o_;f_;g_;s_	0.02	0.1	0.31	1.72	0.83	4.21
p_Proteobacteria;c_Deltaproteobacteria;o_Desulfobacterales;f_Desulfobulbaceae;g_;s_	0.12	0.06	0.29	1.57	0.79	5
p_Proteobacteria;c_Deltaproteobacteria;o_Desulfobacterales;f_Desulfobacteraceae;g_Desulfococcus;s_	0.14	0.14	0.27	1.63	0.73	5.73
p_Proteobacteria;c_Deltaproteobacteria;o_Desulfobacterales;f_Desulfobacteraceae;g_;s_	0.1	0.07	0.26	1.44	0.69	6.42
p_Proteobacteria;c_Deltaproteobacteria;o_;f_;g_;s_	0.15	0.1	0.25	1.35	0.68	7.1
p_Bacteroidetes;c_Cytophagia;o_Cytophagales;f_Flammeovirgaceae;g_;s_	0.15	0.17	0.24	1.43	0.65	7.75
p_Proteobacteria;c_Deltaproteobacteria;o_Desulfuromonadales;f_Desulfuromonadaceae;g_;s_	0.11	0.07	0.24	1.32	0.64	8.39
p_OP8;c_OP8_1;o_;f_;g_;s_	0.07	0.06	0.23	1.3	0.63	9.02
p_Fusobacteria;c_Fusobacteriia;o_Fusobacteriales;f_Fusobacteriaceae;g_Propionigenium;s_	0.07	0.01	0.23	2.65	0.61	9.63
p_Lentisphaerae;c_[Lentisphaeria];o_Lentisphaerales;f_;g_;s_	0.08	0.02	0.23	1.86	0.61	10.24

Groups Mica & Ewing Bank
Average dissimilarity = 35.71

Species	Group Mica Av.Abund	Group Ewing Bank Av.Abund	Av.Diss	Diss/SD	Contrib%	Cum.%
p_Proteobacteria;c_Gammaproteobacteria;o_Thiotrichales;f_Piscirickettsiaceae;g_;s_	0.29	0.27	0.46	1.4	1.28	1.28
p_Chloroflexi;c_Dehalococcoidetes;o_Dehalococcoidales;f_Dehalococcoidaceae;g_;s_	0.09	0.15	0.44	1.32	1.22	2.5
p_Planctomycetes;c_Phycisphaerae;o_MSBL9;f_;g_;s_	0.16	0.16	0.3	1.33	0.85	3.35
p_Proteobacteria;c_Deltaproteobacteria;o_Desulfobacterales;f_Desulfobacteraceae;g_Desulfococcus;s_	0.13	0.14	0.3	1.43	0.85	4.2

p_Bacteroidetes;c_Bacteroidia;o_Bacteroidales;f_;g_;s_	0.12	0.08	0.3	1.43	0.83	5.03
p_Bacteroidetes;c_Cytophagia;o_Cytophagales;f_Flammeovirgaceae;g_;s_	0.21	0.17	0.27	1.36	0.76	5.79
p_Proteobacteria;c_Deltaproteobacteria;o_Desulfobacterales;f_Desulfobulbaceae;g_;s_	0.1	0.06	0.26	1.38	0.73	6.52
p_NC10;c_wb1-A12;o_;f_;g_;s_	0.04	0.1	0.26	1.4	0.73	7.25
p_Proteobacteria;c_Alphaproteobacteria;o_;f_;g_;s_	0.14	0.18	0.23	1.34	0.65	7.9
p_Proteobacteria;c_Deltaproteobacteria;o_Desulfobacterales;f_Desulfobacteraceae;g_;s_	0.07	0.07	0.23	1.57	0.65	8.55
p_OP8;c_OP8_1;o_;f_;g_;s_	0.03	0.06	0.22	1.29	0.6	9.16
p_Proteobacteria;c_Deltaproteobacteria;o_Desulfuromonadales;f_Desulfuromonadaceae;g_;s_	0.09	0.07	0.19	1.36	0.55	9.7

Groups *U-166* & Ewing Bank
Average dissimilarity = 37.69

Species	Group *U-166* Av.Abund	Group Ewing Bank Av.Abund	Av.Diss	Diss/SD	Contrib%	Cum.%
p_Proteobacteria;c_Gammaproteobacteria;o_Thiotrichales;f_Piscirickettsiaceae;g_;s_	0.29	0.27	0.53	1.34	1.39	1.39
p_Chloroflexi;c_Dehalococcoidetes;o_Dehalococcoidales;f_Dehalococcoidaceae;g_;s_	0.08	0.15	0.48	1.32	1.26	2.66
p_Planctomycetes;c_Phycisphaerae;o_MSBL9;f_;g_;s_	0.16	0.16	0.46	1.51	1.22	3.88
p_Proteobacteria;c_Deltaproteobacteria;o_Desulfobacterales;f_Desulfobacteraceae;g_Desulfococcus;s_	0.11	0.14	0.37	1.44	0.99	4.87
p_Bacteroidetes;c_Bacteroidia;o_Bacteroidales;f_;g_;s_	0.11	0.08	0.36	1.43	0.95	5.82
p_Proteobacteria;c_Alphaproteobacteria;o_Rhodobacterales;f_Rhodobacteraceae;g_;s_	0.2	0.12	0.35	1.57	0.93	6.75
p_Actinobacteria;c_OPB41;o_;f_;g_;s_	0.1	0.06	0.27	1.14	0.7	7.45
p_Proteobacteria;c_Alphaproteobacteria;o_;f_;g_;s_	0.19	0.18	0.26	1.4	0.68	8.13
p_OP8;c_OP8_1;o_;f_;g_;s_	0.01	0.06	0.24	1.2	0.63	8.76
p_Bacteroidetes;c_Cytophagia;o_Cytophagales;f_Flammeovirgaceae;g_;s_	0.2	0.17	0.24	1.29	0.62	9.39
p_Proteobacteria;c_Deltaproteobacteria;o_Desulfarculales;f_Desulfarculaceae;g_;s_	0.06	0.1	0.23	1.43	0.6	9.98
p_Proteobacteria;c_Deltaproteobacteria;o_Desulfobacterales;f_Desulfobacteraceae;g_;s_	0.05	0.07	0.22	1.67	0.59	10.57

Groups *Halo* & Viosca Knoll
Average dissimilarity = 31.40

Species	Group *Halo* Av.Abund	Group Viosca Knoll Av.Abund	Av.Diss	Diss/SD	Contrib%	Cum.%
p_Chloroflexi;c_Dehalococcoidetes;o_Dehalococcoidales;f_Dehalococcoidaceae;g_;s_	0.14	0.08	0.35	0.95	1.13	1.13
p_Proteobacteria;c_Gammaproteobacteria;o_Thiotrichales;f_Piscirickettsiaceae;g_;s_	0.25	0.33	0.34	1.28	1.08	2.21
p_Proteobacteria;c_Deltaproteobacteria;o_Desulfobacterales;f_Desulfobacteraceae;g_Desulfococcus;s_	0.14	0.18	0.22	1.53	0.71	2.92
p_Planctomycetes;c_Phycisphaerae;o_MSBL9;f_;g_;s_	0.11	0.15	0.22	1.77	0.7	3.61
p_Proteobacteria;c_Deltaproteobacteria;o_;f_;g_;s_	0.15	0.11	0.21	1.36	0.66	4.27
p_Proteobacteria;c_Deltaproteobacteria;o_Desulfuromonadales;f_Desulfuromonadaceae;g_;s_	0.11	0.06	0.2	1.46	0.64	4.91
p_Lentisphaerae;c_[Lentisphaeria];o_Lentisphaerales;f_;g_;s_	0.08	0.02	0.2	1.9	0.64	5.55
p_Proteobacteria;c_Gammaproteobacteria;o_Oceanospirillales;f_Endozoicimonaceae;g_;s_	0.06	0	0.18	1.33	0.58	6.14
p_OP8;c_OP8_1;o_;f_;g_;s_	0.07	0.03	0.18	0.95	0.57	6.71
p_Proteobacteria;c_Deltaproteobacteria;o_Desulfobacterales;f_Desulfobacteraceae;g_;s_	0.1	0.07	0.18	1.52	0.57	7.28
p_Proteobacteria;c_Deltaproteobacteria;o_Desulfobacterales;f_Desulfobulbaceae;g_;s_	0.12	0.1	0.18	1.49	0.57	7.85
p_Bacteroidetes;c_Bacteroidia;o_Bacteroidales;f_;g_;s_	0.11	0.13	0.17	1.45	0.55	8.4
p_Chloroflexi;c_Dehalococcoidetes;o_;f_;g_;s_	0.07	0.04	0.17	1	0.55	8.95

Groups *U-166* & Viosca Knoll
Average dissimilarity = 34.73

Species	Group *U-166* Av.Abund	Group Viosca Knoll Av.Abund	Av.Diss	Diss/SD	Contrib%	Cum.%
p_Planctomycetes;c_Phycisphaerae;o_MSBL9;f_;g_;s_	0.16	0.15	0.42	2.1	1.2	1.2
p_Proteobacteria;c_Gammaproteobacteria;o_Thiotrichales;f_Piscirickettsiaceae;g_;s_	0.29	0.33	0.37	1.35	1.07	2.27
p_Proteobacteria;c_Deltaproteobacteria;o_Desulfobacterales;f_Desulfobacteraceae;g_Desulfococcus;s_	0.11	0.18	0.37	1.42	1.06	3.33
p_Bacteroidetes;c_Bacteroidia;o_Bacteroidales;f_;g_;s_	0.11	0.13	0.35	1.8	1.02	4.35
p_NC10;c_wb1-A12;o_;f_;g_;s_	0.11	0.03	0.32	2.11	0.93	5.28
p_Chloroflexi;c_Dehalococcoidetes;o_Dehalococcoidales;f_Dehalococcoidaceae;g_;s_	0.08	0.08	0.27	1.47	0.77	6.05
p_Proteobacteria;c_Alphaproteobacteria;o_Rhodobacterales;f_Rhodobacteraceae;g_;s_	0.2	0.14	0.27	1.36	0.76	6.82
p_Proteobacteria;c_Deltaproteobacteria;o_Myxococcales;f_;g_;s_	0.11	0.17	0.24	3.06	0.69	7.5
p_Acidobacteria;c_OS-K;o_;f_;g_;s_	0.04	0.1	0.24	1.53	0.69	8.19
p_Proteobacteria;c_Alphaproteobacteria;o_;f_;g_;s_	0.19	0.17	0.24	2.01	0.68	8.87
p_Actinobacteria;c_OPB41;o_;f_;g_;s_	0.1	0.06	0.23	1.13	0.66	9.53
p_Proteobacteria;c_Deltaproteobacteria;o_Desulfobacterales;f_Desulfobulbaceae;g_;s_	0.06	0.1	0.2	1.54	0.59	10.12

Groups *Anona* & Viosca Knoll
Average dissimilarity = 29.48

Species	Group *Anona* Av.Abund	Group Viosca Knoll Av.Abund	Av.Diss	Diss/SD	Contrib%	Cum.%
p_Proteobacteria;c_Gammaproteobacteria;o_Thiotrichales;f_Piscirickettsiaceae;g_;s_	0.27	0.33	0.35	1.56	1.2	1.2
p_Proteobacteria;c_Deltaproteobacteria;o_Desulfobacterales;f_Desulfobacteraceae;g_Desulfococcus;s_	0.15	0.18	0.29	1.3	1	2.2
p_Bacteroidetes;c_Bacteroidia;o_Bacteroidales;f_;g_;s_	0.14	0.13	0.28	1.76	0.97	3.17
p_Proteobacteria;c_Deltaproteobacteria;o_Desulfobacterales;f_Desulfobulbaceae;g_;s_	0.13	0.1	0.26	1.69	0.88	4.05
p_Planctomycetes;c_Phycisphaerae;o_MSBL9;f_;g_;s_	0.16	0.15	0.26	1.63	0.87	4.92
p_Chloroflexi;c_Dehalococcoidetes;o_Dehalococcoidales;f_Dehalococcoidaceae;g_;s_	0.08	0.08	0.24	1.46	0.82	5.74
p_Proteobacteria;c_Deltaproteobacteria;o_Desulfobacterales;f_Desulfobacteraceae;g_;s_	0.09	0.07	0.19	1.72	0.66	6.4
p_Proteobacteria;c_Alphaproteobacteria;o_;f_;g_;s_	0.17	0.17	0.17	1.82	0.59	6.99
p_Acidobacteria;c_OS-K;o_;f_;g_;s_	0.08	0.1	0.17	1.5	0.59	7.58
p_Bacteroidetes;c_Cytophagia;o_Cytophagales;f_Flammeovirgaceae;g_;s_	0.19	0.17	0.16	1.41	0.53	8.11
p_Proteobacteria;c_Deltaproteobacteria;o_NB1-j;f_JTB38;g_;s_	0.13	0.11	0.16	1.33	0.53	8.64
p_Proteobacteria;c_Deltaproteobacteria;o_Desulfarculales;f_Desulfarculaceae;g_;s_	0.08	0.09	0.15	1.47	0.51	9.15
p_TM6;c_SJA-4;o_;f_;g_;s_	0.1	0.06	0.14	1.16	0.48	9.64
p_Proteobacteria;c_Deltaproteobacteria;o_Syntrophobacterales;f_Syntrophobacteraceae;g_;s_	0.13	0.15	0.13	1.37	0.46	10.1

Groups Ewing Bank & Viosca Knoll

Average dissimilarity = 34.41

Species	Group Ewing Bank Av.Abund	Group Viosca Knoll Av.Abund	Av.Diss	Diss/SD	Contrib%	Cum.%
p_Proteobacteria;c_Gammaproteobacteria;o_Thiotrichales;f_Piscirickettsiaceae;g_;s_	0.27	0.33	0.45	1.31	1.31	1.31
p_Chloroflexi;c_Dehalococcoidetes;o_Dehalococcoidales;f_Dehalococcoidaceae;g_;s_	0.15	0.08	0.43	1.32	1.25	2.56
p_Proteobacteria;c_Deltaproteobacteria;o_Desulfobacterales;f_Desulfobacteraceae;g_Desulfococcus;s_	0.14	0.18	0.32	1.36	0.93	3.49
p_Planctomycetes;c_Phycisphaerae;o_MSBL9;f_;g_;s_	0.16	0.15	0.28	1.32	0.82	4.31
p_NC10;c_wb1-A12;o_;f_;g_;s_	0.1	0.03	0.28	1.51	0.81	5.13
p_Bacteroidetes;c_Bacteroidia;o_Bacteroidales;f_;g_;s_	0.08	0.13	0.26	1.34	0.76	5.88
p_Proteobacteria;c_Deltaproteobacteria;o_Desulfobacterales;f_Desulfobulbaceae;g_;s_	0.06	0.1	0.23	1.37	0.68	6.56
p_Bacteroidetes;c_Cytophagia;o_Cytophagales;f_Flammeovirgaceae;g_;s_	0.17	0.17	0.22	1.48	0.65	7.21
p_Proteobacteria;c_Deltaproteobacteria;o_Desulfobacterales;f_Desulfobacteraceae;g_;s_	0.07	0.07	0.22	1.81	0.64	7.85
p_Acidobacteria;c_OS-K;o_;f_;g_;s_	0.05	0.1	0.21	1.31	0.61	8.46
p_OP8;c_OP8_1;o_;f_;g_;s_	0.06	0.03	0.21	1.32	0.61	9.08
p_Proteobacteria;c_Deltaproteobacteria;o_Sva0853;f_JTB36;g_;s_	0.09	0.05	0.2	1.21	0.58	9.66

Groups *Halo* & Mardi Gras
Average dissimilarity = 40.51

Species	Group *Halo* Av.Abund	Group Mardi Gras Av.Abund	Av.Diss	Diss/SD	Contrib%	Cum.%
p_Proteobacteria;c_Gammaproteobacteria;o_Thiotrichales;f_Piscirickettsiaceae;g_;s_	0.25	0.23	0.48	1.61	1.18	1.18
p_Planctomycetes;c_Phycisphaerae;o_MSBL9;f_;g_;s_	0.11	0.2	0.47	1.9	1.15	2.33
p_Bacteroidetes;c_Bacteroidia;o_Bacteroidales;f_;g_;s_	0.11	0.18	0.39	1.84	0.97	3.31
p_Chloroflexi;c_Dehalococcoidetes;o_Dehalococcoidales;f_Dehalococcoidaceae;g_;s_	0.14	0.1	0.39	1.01	0.97	4.28
p_Proteobacteria;c_Deltaproteobacteria;o_Desulfobacterales;f_Desulfobacteraceae;g_Desulfococcus;s_	0.14	0.19	0.33	1.28	0.81	5.09
p_Proteobacteria;c_Deltaproteobacteria;o_NB1-j;f_NB1-i;g_;s_	0.16	0.22	0.26	1.54	0.64	5.72
p_Lentisphaerae;c_[Lentisphaeria];o_Lentisphaerales;f_;g_;s_	0.08	0.01	0.25	2.17	0.61	6.34
p_Proteobacteria;c_Deltaproteobacteria;o_;f_;g_;s_	0.15	0.1	0.24	1.42	0.6	6.93
p_Actinobacteria;c_OPB41;o_;f_;g_;s_	0.04	0.09	0.24	1.22	0.59	7.53
p_Fusobacteria;c_Fusobacteriia;o_Fusobacteriales;f_Fusobacteriaceae;g_Propionigenium;s_	0.07	0	0.24	2.78	0.59	8.12
p_Bacteroidetes;c_Cytophagia;o_Cytophagales;f_Flammeovirgaceae;g_;s_	0.15	0.17	0.23	1.36	0.58	8.7
p_Proteobacteria;c_Deltaproteobacteria;o_Desulfarculales;f_Desulfarculaceae;g_;s_	0.11	0.1	0.23	1.27	0.58	9.27

Groups Mica & Mardi Gras
Average dissimilarity = 36.63

Species	Group Mica Av.Abund	Group Mardi Gras Av.Abund	Av.Diss	Diss/SD	Contrib%	Cum.%
p_Proteobacteria;c_Gammaproteobacteria;o_Thiotrichales;f_Piscirickettsiaceae;g_;s_	0.29	0.23	0.52	1.56	1.43	1.43
p_Planctomycetes;c_Phycisphaerae;o_MSBL9;f_;g_;s_	0.16	0.2	0.4	1.57	1.08	2.52
p_Proteobacteria;c_Deltaproteobacteria;o_Desulfobacterales;f_Desulfobacteraceae;g_Desulfococcus;s_	0.13	0.19	0.39	1.32	1.05	3.57
p_Bacteroidetes;c_Bacteroidia;o_Bacteroidales;f_;g_;s_	0.12	0.18	0.38	1.33	1.05	4.62
p_Chloroflexi;c_Dehalococcoidetes;o_Dehalococcoidales;f_Dehalococcoidaceae;g_;s_	0.09	0.1	0.3	1.15	0.82	5.44
p_Bacteroidetes;c_Cytophagia;o_Cytophagales;f_Flammeovirgaceae;g_;s_	0.21	0.17	0.26	1.31	0.71	6.15
p_Actinobacteria;c_OPB41;o_;f_;g_;s_	0.06	0.09	0.25	1.34	0.67	6.82
p_Proteobacteria;c_Deltaproteobacteria;o_Desulfarculales;f_Desulfarculaceae;g_;s_	0.07	0.1	0.24	1.06	0.65	7.47
p_Proteobacteria;c_Deltaproteobacteria;o_Desulfobacterales;f_Desulfobulbaceae;g_;s_	0.1	0.12	0.23	1.33	0.63	8.1
p_Proteobacteria;c_Deltaproteobacteria;o_DTB120;f_;g_;s_	0.06	0	0.22	1.68	0.61	8.71
p_Proteobacteria;c_Alphaproteobacteria;o_;f_;g_;s_	0.14	0.16	0.19	1.38	0.53	9.24
p_Proteobacteria;c_Alphaproteobacteria;o_Rhodospirillales;f_Rhodospirillaceae;g_;s_	0.11	0.07	0.19	1.55	0.52	9.76

Groups *U-166* & Mardi Gras
Average dissimilarity = 39.28

Species	Group *U-166* Av.Abund	Group Mardi Gras Av.Abund	Av.Diss	Diss/SD	Contrib%	Cum.%
p_Proteobacteria;c_Gammaproteobacteria;o_Thiotrichales;f_Piscirickettsiaceae;g_;s_	0.29	0.23	0.57	1.37	1.46	1.46
p_Planctomycetes;c_Phycisphaerae;o_MSBL9;f_;g_;s_	0.16	0.2	0.5	1.38	1.28	2.74
p_Bacteroidetes;c_Bacteroidia;o_Bacteroidales;f_;g_;s_	0.11	0.18	0.48	1.37	1.23	3.97
p_Proteobacteria;c_Deltaproteobacteria;o_Desulfobacterales;f_Desulfobacteraceae;g_Desulfococcus;s_	0.11	0.19	0.48	1.32	1.21	5.18
p_NC10;c_wb1-A12;o_;f_;g_;s_	0.11	0.02	0.38	2.16	0.97	6.15
p_Chloroflexi;c_Dehalococcoidetes;o_Dehalococcoidales;f_Dehalococcoidaceae;g_;s_	0.08	0.1	0.34	1.24	0.86	7.01
p_Actinobacteria;c_OPB41;o_;f_;g_;s_	0.1	0.09	0.32	1.32	0.8	7.81
p_Proteobacteria;c_Alphaproteobacteria;o_Rhodobacterales;f_Rhodobacteraceae;g_;s_	0.2	0.14	0.29	1.32	0.75	8.56
p_Proteobacteria;c_Deltaproteobacteria;o_Desulfobacterales;f_Desulfobulbaceae;g_;s_	0.06	0.12	0.29	1.46	0.75	9.31
p_Proteobacteria;c_Deltaproteobacteria;o_Desulfarculales;f_Desulfarculaceae;g_;s_	0.06	0.1	0.28	1.08	0.7	10.01

Groups Ewing Bank & Mardi Gras
Average dissimilarity = 40.72

Species	Group Ewing Bank Av.Abund	Group Mardi Gras Av.Abund	Av.Diss	Diss/SD	Contrib%	Cum.%
p_Proteobacteria;c_Gammaproteobacteria;o_Thiotrichales;f_Piscirickettsiaceae;g_;s_	0.27	0.23	0.61	1.35	1.51	1.51
p_Bacteroidetes;c_Bacteroidia;o_Bacteroidales;f_;g_;s_	0.08	0.18	0.49	1.58	1.21	2.71
p_Chloroflexi;c_Dehalococcoidetes;o_Dehalococcoidales;f_Dehalococcoidaceae;g_;s_	0.15	0.1	0.46	1.32	1.14	3.85
p_Planctomycetes;c_Phycisphaerae;o_MSBL9;f_;g_;s_	0.16	0.2	0.43	1.33	1.07	4.92
p_Proteobacteria;c_Deltaproteobacteria;o_Desulfobacterales;f_Desulfobacteraceae;g_Desulfococcus;s_	0.14	0.19	0.42	1.3	1.04	5.96
p_NC10;c_wb1-A12;o_;f_;g_;s_	0.1	0.02	0.33	1.61	0.82	6.78
p_Proteobacteria;c_Deltaproteobacteria;o_Desulfobacterales;f_Desulfobulbaceae;g_;s_	0.06	0.12	0.33	1.41	0.8	7.58
p_Bacteroidetes;c_Cytophagia;o_Cytophagales;f_Flammeovirgaceae;g_;s_	0.17	0.17	0.28	1.3	0.69	8.27
p_Actinobacteria;c_OPB41;o_;f_;g_;s_	0.06	0.09	0.27	1.36	0.67	8.94
p_Proteobacteria;c_Deltaproteobacteria;o_Desulfarculales;f_Desulfarculaceae;g_;s_	0.1	0.1	0.26	1.24	0.65	9.59
p_Chloroflexi;c_S085;o_;f_;g_;s_	0.11	0.04	0.26	1.43	0.63	10.22

Groups Viosca Knoll & Mardi Gras

Average dissimilarity = 35.18

Species	Group Viosca Knoll Av.Abund	Group Mardi Gras Av.Abund	Av.Diss	Diss/SD	Contrib%	Cum.%
p_Proteobacteria;c_Gammaproteobacteria;o_Thiotrichales;f_Piscirickettsiaceae;g_;s_	0.33	0.23	0.57	1.54	1.61	1.61
p_Planctomycetes;c_Phycisphaerae;o_MSBL9;f_;g_;s_	0.15	0.2	0.39	1.72	1.12	2.73
p_Bacteroidetes;c_Bacteroidia;o_Bacteroidales;f_;g_;s_	0.13	0.18	0.36	1.51	1.04	3.76
p_Proteobacteria;c_Deltaproteobacteria;o_Desulfobacterales;f_Desulfobacteraceae;g_Desulfococcus;s_	0.18	0.19	0.35	1.35	0.99	4.76
p_Chloroflexi;c_Dehalococcoidetes;o_Dehalococcoidales;f_Dehalococcoidaceae;g_;s_	0.08	0.1	0.28	1.13	0.79	5.55
p_Actinobacteria;c_OPB41;o_;f_;g_;s_	0.06	0.09	0.24	1.38	0.67	6.22
p_Proteobacteria;c_Deltaproteobacteria;o_Desulfarculales;f_Desulfarculaceae;g_;s_	0.09	0.1	0.22	1.13	0.64	6.86
p_Proteobacteria;c_Deltaproteobacteria;o_Desulfobacterales;f_Desulfobulbaceae;g_;s_	0.1	0.12	0.2	1.34	0.58	7.43
p_Bacteroidetes;c_Cytophagia;o_Cytophagales;f_Flammeovirgaceae;g_;s_	0.17	0.17	0.2	1.26	0.57	8.01
p_Proteobacteria;c_Gammaproteobacteria;o_Chromatiales;f_;g_;s_	0.13	0.1	0.18	1.83	0.52	8.53
p_Planctomycetes;c_[Brocadiae];o_Brocadiales;f_Brocadiaceae;g_Candidatus Scalindua;s_	0.08	0.05	0.18	1.82	0.5	9.02
p_Chloroflexi;c_Dehalococcoidetes;o_;f_;g_;s_	0.04	0.07	0.18	1.31	0.5	9.52

Table S3. Similarity Percent (SIMPER) analysis, identifying archaeal drivers of within group similarity, and between group dissimilarity.

Group *Halo*
Average similarity: 70.69

Species	Av.Abund	Av.Sim	Sim/SD	Contrib%	Cum.%
p_Crenarchaeota;c_Thaumarchaeota;o_Cenarchaeales;f_Cenarchaeaceae;g_Nitrosopumilus;s_	0.64	16.78	1.88	23.74	23.74

Group Mica
Average similarity: 74.44

Species	Av.Abund	Av.Sim	Sim/SD	Contrib%	Cum.%
p_Crenarchaeota;c_Thaumarchaeota;o_Cenarchaeales;f_Cenarchaeaceae;g_Nitrosopumilus;s_	0.74	23.72	2.76	31.87	31.87

Group *U-166*
Average similarity: 64.57

Species	Av.Abund	Av.Sim	Sim/SD	Contrib%	Cum.%
p_Crenarchaeota;c_Thaumarchaeota;o_Cenarchaeales;f_Cenarchaeaceae;g_Nitrosopumilus;s_	0.68	21.98	1.62	34.04	34.04

Group *Anona*
Average similarity: 67.07

Species	Av.Abund	Av.Sim	Sim/SD	Contrib%	Cum.%
p_Crenarchaeota;c_Thaumarchaeota;o_Cenarchaeales;f_Cenarchaeaceae;g_Nitrosopumilus;s_	0.7	20.16	2.1	30.05	30.05

Group Ewing Bank
Average similarity: 69.01

Species	Av.Abund	Av.Sim	Sim/SD	Contrib%	Cum.%
p_Crenarchaeota;c_Thaumarchaeota;o_Cenarchaeales;f_Cenarchaeaceae;g_Nitrosopumilus;s_	0.65	19.57	2.29	28.35	28.35

Group Viosca Knoll
Average similarity: 77.82

Species	Av.Abund	Av.Sim	Sim/SD	Contrib%	Cum.%
p_Crenarchaeota;c_Thaumarchaeota;o_Cenarchaeales;f_Cenarchaeaceae;g_Nitrosopumilus;s_	0.78	25.52	3.35	32.8	32.8

Group Mardi Gras
Average similarity: 58.83

Species	Av.Abund	Av.Sim	Sim/SD	Contrib%	Cum.%
p_Crenarchaeota;c_Thaumarchaeota;o_Cenarchaeales;f_Cenarchaeaceae;g_Nitrosopumilus;s_	0.57	14.32	1.36	24.34	24.34

Groups *Halo* & Mica
Average dissimilarity = 35.56

	Group Halo	Group Mica				
Species	Av.Abund	Av.Abund	Av.Diss	Diss/SD	Contrib%	Cum.%
p_Crenarchaeota;c_MCG;o_;f_;g_;s_	0.4	0.22	4.39	1.4	12.35	12.35

Groups *Halo* & *U-166*
Average dissimilarity = 43.24

	Group Halo	Group U-166				
Species	Av.Abund	Av.Abund	Av.Diss	Diss/SD	Contrib%	Cum.%
p_Crenarchaeota;c_MCG;o_;f_;g_;s_	0.4	0.17	5.12	1.41	11.84	11.84

Groups Mica & *Anona*
Average dissimilarity = 28.42

	Group Mica	Group Anona				
Species	Av.Abund	Av.Abund	Av.Diss	Diss/SD	Contrib%	Cum.%
p_Crenarchaeota;c_MCG;o_;f_;g_;s_	0.22	0.28	3.74	1.48	13.18	13.18

Groups *Halo* & Ewing Bank
Average dissimilarity = 36.28

	Group Halo	Group Ewing Bank				
Species	Av.Abund	Av.Abu	Av.Diss	Diss/SD	Contrib%	Cum.%
p_Crenarchaeota;c_MCG;o_;f_;g_;s_	0.4	0.26	4.38	1.37	12.08	12.08

Groups *Halo* & Mardi Gras
Average dissimilarity = 43.89

	Group Halo	Group Mardi Gras				
Species	Av.Abund	Av.Abu	Av.Diss	Diss/SD	Contrib%	Cum.%
p_Crenarchaeota;c_Thaumarchaeota;o_Cenarchaeales;f_Cenarchaeaceae;g_Nitrosopumilus;s_	0.64	0.57	5.01	1.4	11.42	11.42

Groups Mica & Mardi Gras

Average dissimilarity = 38.07

Species	Group Mica Av.Abund	Group Mardi Gras Av.Abu	Av.Diss	Diss/SD	Contrib%	Cum.%
p_Crenarchaeota;c_Thaumarchaeota;o_Cenarchaeales;f_Cenarchaeaceae;g_Nitrosopumilus;s_	0.74	0.57	5.03	1.4	13.22	13.22

Groups *U-166* & Mardi Gras
Average dissimilarity = 44.81

Species	Group U-16(Av.Abund	Group Mardi Gras Av.Abu	Av.Diss	Diss/SD	Contrib%	Cum.%
p_Crenarchaeota;c_Thaumarchaeota;o_Cenarchaeales;f_Cenarchaeaceae;g_Nitrosopumilus;s_	0.68	0.57	5.25	1.28	11.71	11.71

Groups Ewing Bank & Mardi Gras
Average dissimilarity = 41.78

Species	Group Ewing Av.Abu	Group Mardi Gras Av.Abu	Av.Diss	Diss/SD	Contrib%	Cum.%
p_Crenarchaeota;c_Thaumarchaeota;o_Cenarchaeales;f_Cenarchaeaceae;g_Nitrosopumilus;s_	0.65	0.57	4.71	1.43	11.27	11.27

Groups Viosca Knoll & Mardi Gras
Average dissimilarity = 36.51

Species	Group Viosc Av.Ab	Group Mardi Gras Av.Abu	Av.Diss	Diss/SD	Contrib%	Cum.%
p_Crenarchaeota;c_Thaumarchaeota;o_Cenarchaeales;f_Cenarchaeaceae;g_Nitrosopumilus;s_	0.78	0.57	5.15	1.41	14.1	14.1
